# Phyto-mediated metallic nano-architectures *via Melissa officinalis* L.*:* synthesis, characterization and biological properties

**DOI:** 10.1038/s41598-017-12804-7

**Published:** 2017-09-29

**Authors:** Irina Fierascu, Milen I. Georgiev, Alina Ortan, Radu Claudiu Fierascu, Sorin Marius Avramescu, Daniela Ionescu, Anca Sutan, Alexandru Brinzan, Lia Mara Ditu

**Affiliations:** 1 University of Agronomic Science and Veterinary Medicine, 59 Marasti Blvd, 011464 Bucharest, Romania; 2 The National Institute for Research & Development in Chemistry and Petrochemistry-ICECHIM, 202 Spl. Independentei, 060021 Bucharest, Romania; 3 Laboratory of Applied Biotechnologies, Institute of Microbiology, Bulgarian Academy of Sciences, 139 Ruski Boulevard, 4000 Plovdiv, Bulgaria; 4 University of Bucharest, Faculty of Chemistry, PROTMED Research center, 36-46M. Kogalniceanu Blvd., 050107 Bucharest, Romania; 5 S.C. HOFIGAL EXPORT IMPORT S.A., 2 Intrarea Serelor, 042124, sector 4, Bucharest, Romania; 6 University of Pitesti, Faculty of Science, 1 Targu din Vale Str, 110040 Pitesti, Romania; 7 Romanian Academy, Institute of Biology – Bucharest, 296 Spl. Independentei, 060031 Bucharest, Romania; 8 University of Bucharest, Microbiology Department, 1-3 Aleea Portocalelor, 060101 Bucharest, Romania

## Abstract

The development of methods for obtaining new materials with antimicrobial properties, based on green chemistry principles has been a target of research over the past few years. The present paper describes the phyto-mediated synthesis of metallic nano-architectures (gold and silver) *via* an ethanolic extract of *Melissa officinalis* L. (obtained by accelerated solvent extraction). Different analytic methods were applied for the evaluation of the extract composition, as well as for the characterization of the phyto-synthesized materials. The cytogenotoxicity of the synthesized materials was evaluated by *Allium cepa* assay, while the antimicrobial activity was examined by applying both qualitative and quantitative methods. The results demonstrate the synthesis of silver nanoparticles (average diameter 13 nm) and gold nanoparticles (diameter of ca. 10 nm); the bi-metallic nanoparticles proved to have a core-shell flower-like structure, composed of smaller particles (ca. 8 nm). The Ag nanoparticles were found not active on nuclear DNA damage. The Au nanoparticles appeared nucleoprotective, but were aggressive in generating clastogenic aberrations in *A. cepa* root meristematic cells. Results of the antimicrobial assays show that silver nanoparticles were active against most of the tested strains, as the lowest MIC value being obtained against *B. cereus* (approx. 0.0015 mM).

## Introduction

The conventional chemical methods for obtaining metallic nanoparticles with organic solvents, toxic chemicals and non-biodegradable stabilizing agents are no longer a trend for the future research^1^. In order to obtain materials with controlled size and morphology, as well as with certain properties, research efforts are nowadays focused on the border areas of science, combining knowledge from chemistry, biology and nanotechnology^2–5^. In addition, because both the number of severe infection diseases and the increase in resistance of most pathogens to the available drugs are steadily increasing nowadays, another challenge in this area is the discovery of new materials that can be used as drugs^3^.

Due to their abilities to damage the bacterial DNA, cell membrane and critical enzymes, noble metal nanoparticles are considered to be promising antibacterial agents^6,7^. In a very close correlation with their content in bioactive molecules, plant extracts have been successfully used to generate noble metal naoparticles^8–12^. Among the noble metals, silver is the most common and widely applied as an antibacterial agent^13^. Gold nanoparticles can also be found in a wide range of applications, from imaging to drug delivery thermotherapy^14^. Regarding their cytotoxicity, silver nanoparticles were found to have a relatively high toxicity^15^, gold nanoparticles are inert^16^, while the gold/silver bi-metallic nanoparticles have an intermediary biologic action^17,18^.

*Melissa officinalis* L., commonly known as lemon balm, is a well-known medicinal plant, belonging to the Lamiaceae family, with a strong specific scent; it is commonly used in folk medicine of many countries. Since ancient times, when plants were used as teas, decoctions or they were used as such, *Melissa officinalis* L. was used for the treatment of mental diseases, cardiovascular and respiratory problems, as memory enhancer, antidepressant, sleeping aid and antidote^19,20^. Recent studies have shown its antihyperlipidemic, anti-inflammatory and antioxidant properties^21–23^. Besides the pharmaceutical use, other industrial applications of *M. officinalis* are related to its use in apiculture (lemon balm being also known as *bee balm* or *honey balm* ^24^, in food and liquors industries, in cosmetics or as ornaments^20^. Also, it has been revealed that the *M. officinalis* leaf extract possesses antiviral properties due to the content of phenolic acids, while its essential oil has antibacterial, antifungal, and antihistaminic activities because of constituents such as geranial, neral, (E)-anethole, (E)-caryophyllene, and citronellal^25–27^.

Literature data^20^ shows a high content of phytochemicals (known to be involved in the phytosynthesis of metallic nanoparticles, including terpenes and phenolic compounds) in the *M. officinalis* extracts; this, in turn, suggests a very high potential for the synthesis of metallic nanoparticles, an application of *M. officinalis* not sufficiently explored in the published literature data.

The present manuscript describes the phyto-mediated synthesis of metallic nano-architectures (gold, silver and gold/silver bimetallic nanoparticles) *via* the ethanolic extract of *M. officinalis* (obtained by accelerated solvent extraction). Analytic methods (XRD, XRF, UV-Vis, electron microscopy, chromatographic techniques – GC-MS, HPLC) were applied for the evaluation of the extract composition, as well as for the characterization of the phyto-synthesized materials. The cytotoxicity of the synthesized materials was evaluated in *Allium cepa* assay, while the antimicrobial activity was evaluated by using both qualitative and quantitative methods. Qualitative screening was performed by diffusion method (adapted from CLSI standard methods) and quantitative analysis was performed by binary serial micro-dilution method in liquid medium, in order to determine the Minimal Inhibitory Concentration values. Microbial strains included in the present study belong to different genera and species (molds, yeast and bacteria strains).

## Materials and Methods

### Vegetal material, extraction procedure and metal nanoparticle phytosynthesis

Leaves of certified *M. officinalis* L. were harvested at full matureness stage from the ecological crops of S.C. HOFIGAL Export Import S.A (Bucharest, Romania). The plants were shade dried and grinded to powder. The extract was obtained using an accelerated solvent extractor ASE 350 (Dionex, Thermo Scientific), equipped with a solvent controller unit. The dried plant samples (5 g) were mixed with silica beads and packed into 34 mL cells using ethanol as an extraction solvent. Prior to solvent extraction an initial heat-up step was applied. Static extractions were performed and the cell was rinsed with the selected solvent; the solvent was purged from the cell using N_2_ gas. The extraction conditions were as follows: temperature: 100 °C; pressure: 1500 psi; static time: 5 min; static cycles: 1; flush volume: 60%; purge time: 120 s. The extraction procedure was selected considering our previous results regarding nanoparticle synthesis that showed that more efficient extraction procedure leads to smaller and more active nanoparticles^4^.

The phytosynthesis of the metallic nanoparticles was accomplished using a source of metallic ions (AgNO_3_ and HAuCl_4_ × 3H_2_O; Merck KGaA, Germany) and ethanolic extract of *M. officinalis* as reducing agent. The general recipe involves the drop-wise addition of 10 mL extract in 40 mL 10^−3^M metal containing solution, under vigorous stirring. For the synthesis of bi-metallic nanoparticles, the metals (Ag and Au) were in equal molar concentration (0.5 × 10^−3^ M).

### Analytical characterization methods

The obtained extract was characterized by means of GC-MS and HPLC, in order to evaluate the extract phytochemical composition. The GC-MS analysis was carried out using a 7010 GC-MS triple Quad system (Agilent, USA). The system is equipped with a HP-5MS Inert column with dimensions of 30 mm × 0.25 mm ID × 0.25 μm. The carrier gas used is helium with a flow of 1.0 mL min^−1^. The injector was operated at 250 °C and the oven temperature was programmed as follows: 50 °C for 2 min, then gradually increased to 290 °C at 5 °C min^−1^. The identification of compounds was based on Flavor2 and NIST14 libraries as well as comparison of their retention indexes.

The HPLC analyses were performed on a Varian system (solvent delivery pumps Prostar 410, DAD detector Prostar 335 and autosampler Prostar 410); obtained data were analyzed using Varian Workstation 6.3 software. The mobile phase consisted of two different solutions, solution A (water, acidified with 1% CH_3_COOH) and solution B (acetonitrile, acidified with 1% CH_3_COOH). The flow rate was 1 mL min^−1^ with an injection volume of 10 μL. Calibration curves were constructed for each of the analyzed compounds (R^2^ > 0.999), using commercial available standards: phenolic acids (chlorogenic acid, ferulic acid, gallic acid and rosmarinic acid), flavonoids (rutin, quercetin and apigenin) and phenylpropenes (*trans*-anethole; all supplied by Merck KGaA, Germany).

Both formation and stability of the nanoparticles were monitored by UV-VIS spectrometry, using a SPECORD 210 Plus UV-VIS Spectrometer, in the wavelength range of 370-450 nm for silver, 500-600 nm for gold and 370-600 nm for gold/silver, respectively. The measurements were performed every four hours, for a period of six days.

X-Ray diffraction measurements were performed using a Rigaku SmartLab equipment, operating at 45 kV and 200 mA, Cu Kα radiation (1.54059 Å), parallel beam configuration (2θ/θ scan mode), from 25 to 90 2θ degrees; the components were identified using the Rigaku Data Analysis Software PDXL 2, database provided by ICDD.

The X-Ray fluorescence measurements were performed in order to evaluate the synthesis of nanoparticles, using an energy-dispersive spectrometer, EDXRF PW4025, type MiniPal 2 (PANalytical, B.V., The Netherlands), with a Si-PIN detector, at 20 kV and automatic current intensity, measurement time 300 seconds, in Helium atmosphere. Aluminum and molybdenum filters were used for the analysis of silver and silver/gold nanoparticles, respectively gold nanoparticles, in order to remove the Rh lines (from the X-ray tube) and other light elements. The Transmission Electron Microscopy images were recorded using a JEOL JEM 1400 electron microscope (80 kV), using copper grids.

### Cytogenotoxicity test

Bulbs of common onion *Allium cepa* L. were purchased from a local market and used as indicator plant, being carefully cleaned without destroying the primordial roots. Bulbs were selected for their phytosanitary status. Three bulbs were used in each experimental group depending on the types and concentration of extracts, distilled water being used as a negative control.

The toxicity assay was performed as a 96-h static exposure at 10% and 20% concentrations of ethanolic extract of *M. officinalis* (R), ethanolic extract of *M. officinalis* with Ag nanoparticles, (S1), ethanolic extract of *M. officinalis* with Au nanoparticles (S2), and ethanolic extract of *M. officinalis* with Ag and Au nanoparticles (S3). The onion bulbs were grown in distilled water for the first 48 h and then transferred for another 48 hours in containers containing test extract and their nanoparticles, respectively.

Root tips at a length of 10 mm were cut off and placed for 24 hours in 3:1 ratio glacial acetic acid: ethanol. 5 root tips for each sample were heated 15 min at 60 **°**C in hydrochloric acid solution 1 N. Fixed and macerated root tips were then stained with aceto-orcein solution 1% for 15 min at 60 **°**C. The roots were placed on a slide in a drop of acetic-water (glacial acetic acid 45%) and the terminal root tips were used to made slides via squash technique. The coded slides were sealed with a few coats of nail varnish, and refrigerated.

All slides were examined using Olympus CX-31 microscope, at 400× magnification. The microscopic analysis was focused on scoring of mitosis phases, chromosome aberrations and nuclear abnormalities, per 3000 scored cells for each sample. The mitotic index was determined as number of mitotic cells among the total amount of scored cells being shown as a percentage^28^. The number of cells at different mitotic stages (prophase, metaphase, anaphase and telophase) was calculated as percentage of the number of dividing cells. The frequency of chromosome aberrations and nuclear abnormalities were calculated as percentage of the number of dividing cells in the appropriate mitotic stage and cell cycle phase, respectively.

The data were analyzed for statistical significance using analysis of variance (one-way ANOVA), and Duncan test was used to determine significant differences among means. Significant differences were set at P = 0.05. Results are presented as the Mean ± standard error of more independent experiments.

### Antimicrobial tests

The antimicrobial assay was performed using the following standard strains: *Staphylococcus aureus* (ATCC 25923), *Bacillus cereus* (B1079), *Pseudomonas aeruginosa* (ATCC 27853), *Escherichia coli* (ATCC 25922), *Candida albicans* (ATCC 10231), *C. parapsilosis* (ATCC 22109), *C. glabrata* (ATCC 64677), *C. krusei* (ATCC 14243), *Aspergillus niger* (strain isolated from soil), *Trichoderma viride* (strain isolated from soil).

An adapted spot diffusion method was followed for the qualitative screening^29^. For the experiments, yeast suspensions of 1.5 × 10^8^ CFU mL^−1^ (0.5 McFarland density) were obtained from cultures developed on yeast extract peptone dextrose solid media. For the filamentous fungi experiments, the density of the spore suspension in phosphate buffer saline (PBS), supplemented with 1 µl mL^−1^ Tween 20 was 0.4–5 × 10^4^ CFU mL^−1^. Petri dishes with YPG (for yeasts) or PDA (potato dextrose agar) (for filamentous fungi) were seeded with fungal inoculums, and an amount of 10 mL solution of each compound of 10 mg mL^−1^ concentration was spotted after seeding. DMSO was used as negative control. After inoculation, the plates were initially kept at room temperature (in order to ensure the equal diffusion of the compound in the medium) and then incubated at 37 °C (for clinical strains) or room temperature (for reference strains) for 24–48 hrs (yeasts) or 5–7 days (for filamentous fungi). The positive results were read as the occurrence of an inhibition zone of fungal growth around the spot.

For the quantitative screening, a binary serial microdilution technique using 96-well microtitre plates was used to obtain the MIC values of the tested compounds against the various microorganisms^30^. The quantitative assay was performed in YPG broth medium for yeasts, Muller Hinton broth for bacteria, and RPMI for filamentous fungi, according with “Performance Standard for Antimicrobial Susceptibility Testing”^31^ and “Reference Method for Broth Dilution Antifungal Susceptibility Testing of Filamentous Fungi; Approved Standard”^32^.

For establishing the MIC (minimum inhibitory concentration) values, a microdilution method performed in nutritive broth was used. The sterile broth was added in sterile 96 well plates and binary dilutions of each tested compound were performed in a final volume of 150 μL. After preparing the binary dilutions, 15 μL of microbial suspension adjusted to an optical density of 0.5 McFarland (1,5 × 10^8^ CFU mL^−1^) were added in each well. The MIC values were established by visual analysis and spectrophotometric measurement (absorbance reading at 600 nm). Each experiment was performed in triplicate and repeated on at least three separate occasions.

For the assessment of adherence on the inert substratum, 96-multi well plastic plates containing binary dilutions of the tested compounds in a final volume of 200 µL were inoculated with 50 µL microbial suspensions of 10^7^ CFU mL^−1^ prepared in sterile saline and inoculated and incubated for 24 h at 37 °C. After incubation, the wells were discarded, washed three times by PBS and the bacterial cells adhered to the plastic walls were stained by 1% violet crystal solution for 15 min. The dyed biofilm was thereafter fixed by cold methanol for 5 minutes and re-suspended by 33% acetic acid solution. The absorbance at 490 nm of the blue suspension was measured using BIOTEK SYNERGY-HTX ELISA multi-mode reader, the obtained values being proportional with the number of the adhered microbial cells.

## Results

Several chromatographic techniques (Table 1 – results of the GC-MS analysis, chromatogram presented in Supplementary Figure S1; Table 2 – results of HPLC analysis, HPLC chromatogram presented in Supplementary Figure S2) were applied in order to analyze the phytochemical composition of the obtained extract.

**Table 1:** Major and minor compounds identified by GC-MS in the *Melissa officinalis* L. extract.

No.	Compound	RT (min.)	Area (%)
1	N-Benzyloxycarbonyl-dl-norleucine	13.517	0.9414
2	Benzyl methanoate	13.769	0.0377
3	Methyl N-(N-benzyloxycarbonyl-beta-l-aspartyl)-beta-d-glucosaminide	14.158	0.0423
4	dl-Leucine, N-[(phenylmethoxy)carbonyl]-	14.283	0.0205
5	(1 R)-(-)-Myrtenal	14.395	0.2137
6	Benzyl isopentyl ether	14.832	0.3334
7	Pentanedioic acid, dimethyl ester	15.396	0.6908
8	4H-Pyran-4-one, 2,3-dihydro-3,5-dihydroxy-6-methyl-	15.626	0.4311
9	Isopulegol	15.776	0.3918
10	3,7-Dimethyl-7-octenal	15.874	0.7839
11	2,5-Octadecadiynoic acid, methyl ester	17.187	0.2840
12	N-Benzyl-2-phenethylamine	17.609	0.9521
13	4-Hepten-3-one, 4-methyl-	17.891	1.3241
14	R-Limonene	18.029	0.1368
15	Hexanedioic acid, dimethyl ester	18.345	1.1701
16	Z,Z,Z-4,6,9-Nonadecatriene	18.653	0.4412
17	2-Methoxy-4-vinylphenol	20.321	0.4115
18	Caryophyllene	23.188	1.5957
19	(3-Nitrophenyl) methanol, isopropyl ether	23.438	10.3997
20	Pyrrolizin-1,7-dione-6-carboxylic acid, methyl(ester)	23.543	0.1999
21	β-Hydroxylauric acid	23.822	0.0424
22	2(4 H)-Benzofuranone, 5,6,7,7a-tetrahydro-4,4,7a-trimethyl-, (R)-	24.714	0.3815
23	Unidentified	26.043	0.5917
24	Caryophyllene oxide	27.158	0.5790
25	[1,1′-Bicyclopropyl]-2-octanoic acid, 2′-hexyl-, methyl ester	27.288	0.0832
25	2-Myristynoyl pantetheine	27.563	0.1947
26	Cyclopropanetetradecanoic acid, 2-octyl-, methyl ester	27.709	0.5649
27	Agaricic acid	28.191	0.1677
28	11,13-Dihydroxy-tetradec-5-ynoic acid, methyl ester	28.394	0.5483
29	10-Heptadecen-8-ynoic acid, methyl ester, (E)-	28.515	0.1399
30	6-epi-shyobunol	28.662	0.1875
31	Cyclopropanetetradecanoic acid, 2-octyl-, methyl ester	28.784	0.4707
32	7-Methyl-Z-tetradecen-1-ol acetate	28.838	1.5319
33	2-[4-methyl-6-(2,6,6-trimethylcyclohex-1-enyl)hexa-1,3,5-trienyl]cyclohex-1-en-1-carboxaldehyde	29.381	0.2547
34	9-Hexadecenoic acid	30.653	0.2862
35	7-Methyl-Z-tetradecen-1-ol acetate	30.972	0.0883
36	Cyclopent-2-ene-1-carboxylic acid, 2,3-dimethyl-1-ethyl-, ethyl ester	31.071	7.1930
37	Unidentified	31.237	0.0216
38	10-Heptadecen-8-ynoic acid, methyl ester, (E)-	31.486	0.7463
39	2-Methoxybenzoic acid, benzyl ester	31.615	0.7083
40	p-Hydroxycinnamic acid, ethyl ester	32.066	0.2478
41	Unidentified	32.150	0.5987
42	Neophytadiene	32.345	11.5269
43	1.Hexa-hydro-farnesol	32.469	0.6797
44	9-Octadecenoic acid, (2-phenyl-1,3-dioxolan-4-yl)methyl ester, trans-	32.754	0.1129
45	Phytol, acetate	32.834	1.9646
46	Phthalic acid, butyl undecyl ester	33.008	0.4107
47	3,7,11,15-Tetramethyl-2-hexadecen-1-ol	33.197	3.5572
48	Palmitic acid	34.711	7.1208
49	Unidentified	35.005	0.4174
50	9-Octadecenoic acid, (2-phenyl-1,3-dioxolan-4-yl)methyl ester, cis-	35.164	0.5406
51	Hexadecanoic acid, ethyl ester	35.335	1.4563
52	Caffeic acid	36.727	1.5472
53	Unidentified	36.779	0.4635
54	Phytol	37.538	7.7461
55	13-Heptadecyn-1-ol	37.863	1.3360
56	Linolenic acid	38.002	7.1616
57	Oleic Acid	38.337	0.7987
58	2-cis-9-Octadecenyloxyethanol	38.393	0.7430
59	Linolenic acid, ethyl ester	38.523	0.8939
60	3-(Benzyloxymethyl)hex-5-ene-1,2-diol	39.362	0.2389
61	Unidentified	40.453	0.4621
62	1-Heptatriacotanol	41.718	0.2889
63	Benzenepropionic acid, 4-benzyloxy-	42.300	3.2737
64	5β)Pregnane-3,20β-diol, 14α,18α-[4-methyl-3-oxo-(1-oxa-4-azabutane-1,4-diyl)]-, diacetate	42.784	0.3836
65	Dipalmitin	44.034	0.5934
66	Diisooctyl phthalate	44.689	5.0982
67	5H-Cyclopropa[3,4]benz[1,2-e]azulen-5-one, 2,9,9a-tris(acetyloxy)-3-[(acetyloxy)methyl]-1,1a,1b,2,4a,7a,7b,8,9,9a-decahydro-4a,7b-dihydroxy- 1,1,6,8-tetramethyl-, [1aR-(1aa,1bb,2b,4ab,7aa,7ba,8a,9b,9aa)]-	44.972	0.3698
68	1H-Cyclopropa[3,4]benz[1,2-e]azulene-5,7b,9,9a-tetrol, 1a,1b,4,4a,5,7a,8,9-octahydro-3-(hydroxymethyl)-1,1,6,8-tetramethyl-, 5,9,9a-triacetate, [1aR-(1aa.,1bb.,4ab.,5.b.,7aa., 7b.a.,8.a.,9.b.,9a.a.)]-	46.126	0.2082
69	4H-Cyclopropa[5′,6′]benz[1′,2′:7,8]azuleno[5,6-b]oxiren-4-one, 8-(acetyloxy)-1,1a,1b,1c,2a,3,3a,6a,6b,7,8,8a-dodecahydro-3a,6b,8a-trihydroxy-2a-(hydroxymethyl)-1,1,5,7-tetramethyl-, [1ar-(1aa.,1bb.,1 c.a.,2a.a.,3a.b.,6a.a.,6b.a.,7.a.,8.b.,8a.a.)]-	46.692	0.1942
70	1-Heptatriacotanol	46.818	1.0470
71	4,9,9a-Tris(acetyloxy)-3-[(acetyloxy)methyl]-1,1a,1b,4,4a,7a,7b,8,9,9a-decahydro-4a,7b-dihydroxy-1,1,6,8-tetramethyl-5H-cyclopropa[3,4]benz[1,2-e]azulen-5-one	47.023	0.4588
71	Unidentified	47.533	0.4993
72	1H-Cyclopropa[3,4]benz[1,2-e]azulene-5,7b,9,9a-tetrol, 1a,1b,4,4a,5,7a,8,9-octahydro- 3-(hydroxymethyl)-1,1,6,8-tetramethyl-, 5,9,9a-triacetate, [1aR-(1a.aa.,1b.b.,4a.b., 5.b.,7a.a.,7b.a.,8.a.,9.b.,9a.a.)]-	48.201	0.1081
73	Ethyl iso-allocholate	48.683	0.2764
74	Unidentified	48.952	0.2799
75	Octadecane, 3-ethyl-5-(2-ethylbutyl)-	49.551	1.3124

Where: RT – retention time (in minutes), area (%) – percent of the total area of the chromatogram.

**Table 2:** HPLC quantification of eight known marker compounds of *Melissa officinalis* L.

No.	Compound	Content (mg/kg), expressed on dry weight basis
1	Gallic acid	7.438 ± 0.014
2	Chlorogenic acid	72.529 ± 0.24
3	Ferulic acid	45.489 ± 0.15
4	Rosmarinic acid	3502.399 ± 1.78
5	Quercetin	153.465 ± 0.32
6	Apigenin	84.538 ± 0.11
7	Rutin	1462.997 ± 1.24
8	trans-Anethole	85.876 ± 0.12

**Figure 1:**
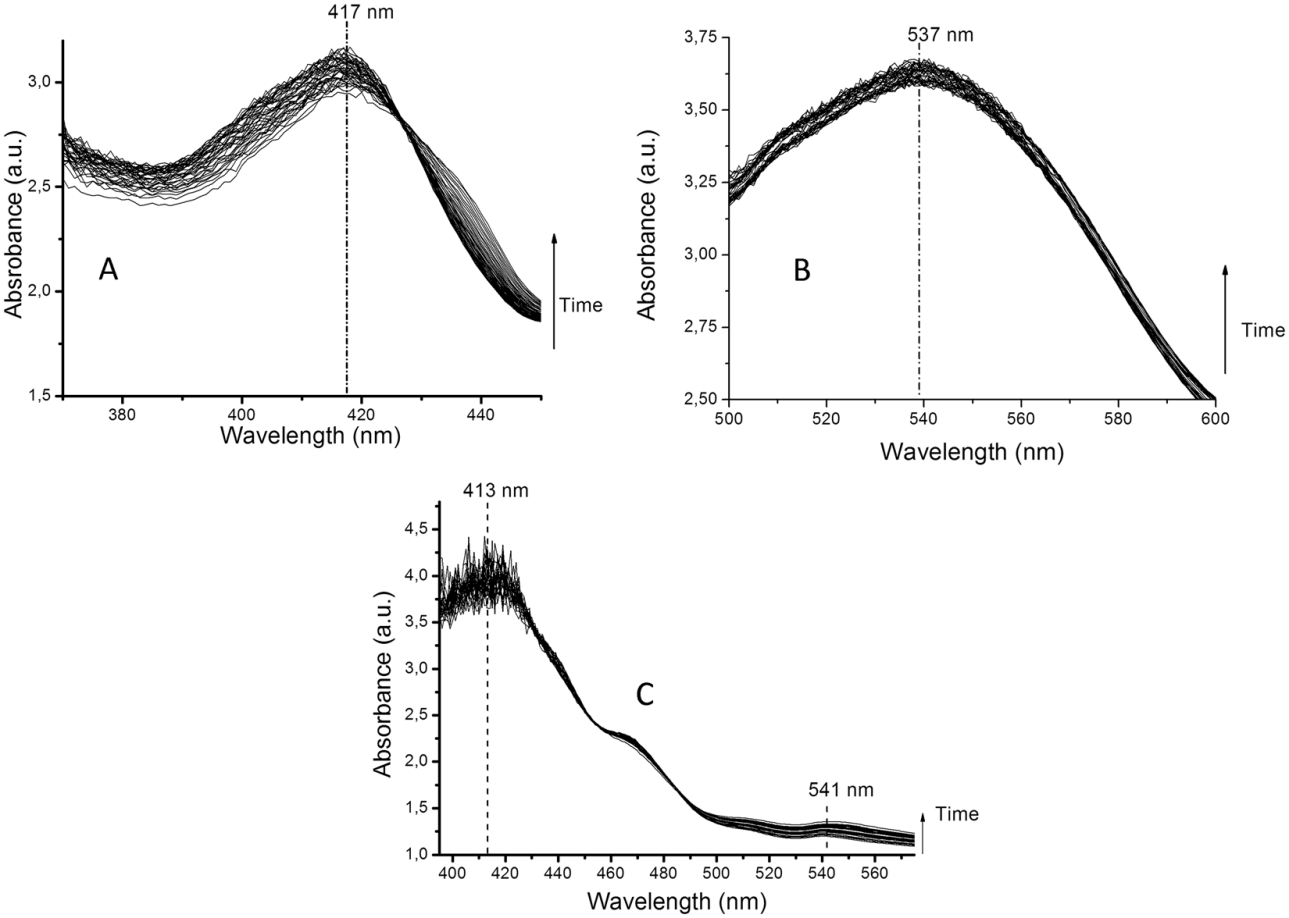
UV-Vis spectra of the synthesized nanoparticles (**A** – silver nanoparticles, **B** – gold nanoparticles, **C** – silver/gold nano-architectures) and their stability in time. The characteristic peaks of silver and gold nanoparticles are presented.

In order to evaluate the synthesis and the stability of the mono and bi-metallic architectures, the UV-Vis spectra were recorded from the reaction medium as a function of a time (every four hours, over six days period, in order to evaluate their stability) (Fig. 1). The analysis focused in the wavelength range of 370–450 nm for silver, 500–600 nm for gold and 370–600 nm for gold/silver. Peaks were recorded at 417 nm for silver, 537 nm for gold and two peaks (at 413 nm and 541 nm) for the bimetallic nanoparticles, respectively.

**Figure 2:**
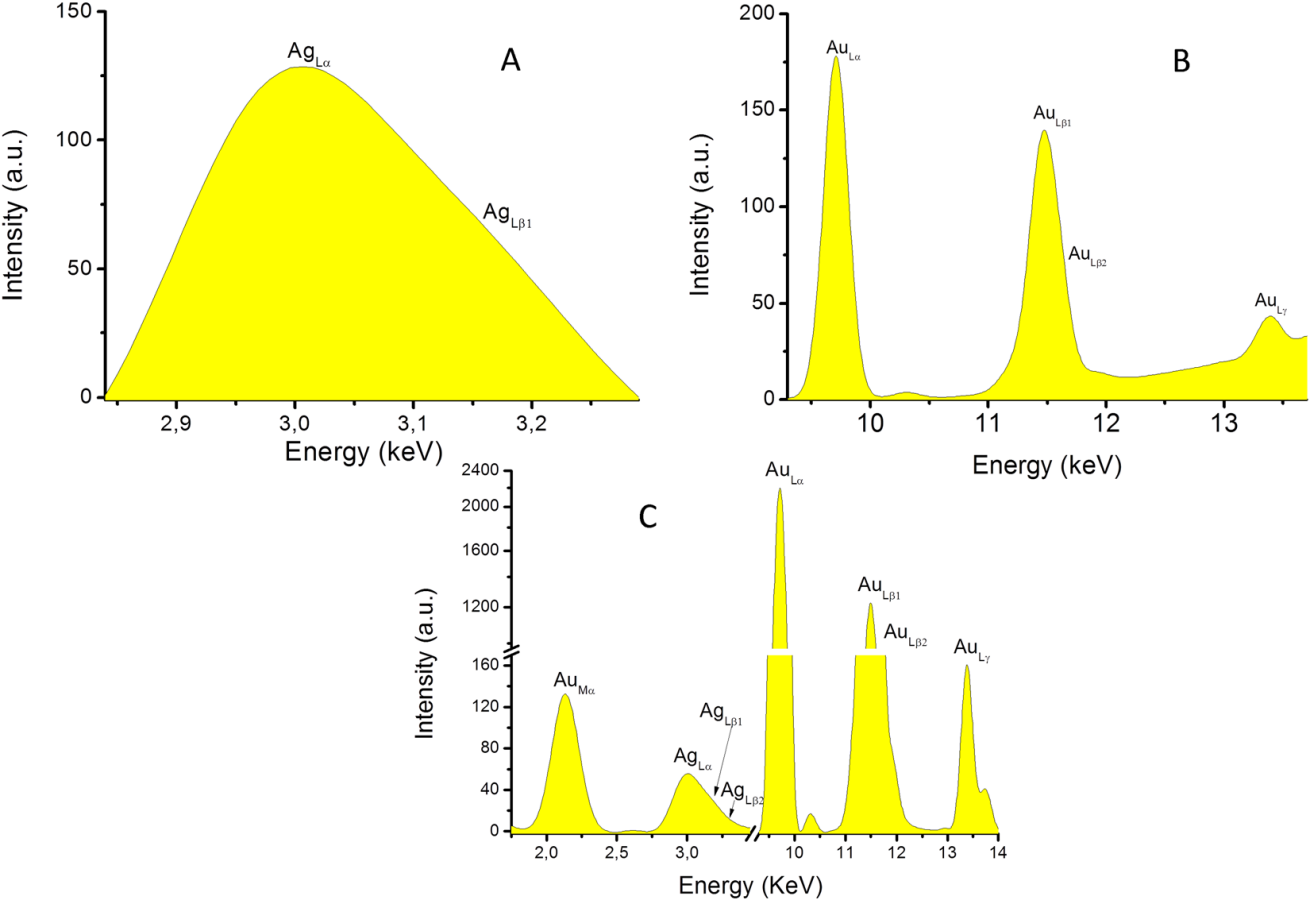
Energy-dispersive X-ray fluorescence spectra of the phytosynthesized nanoparticles (**A** – silver nanoparticles, **B** – gold nanoparticles, **C** – silver/gold nano-architectures). Specific peaks of gold and silver are presented.

For the XRD and XRF analyses, the samples were centrifuged for 60 minutes at 6000 rpm and the sediments containing nanoparticles were dried (for the X-ray diffraction directly on the sample plates and for X-ray fluorescence on a cellulosic support). The XRF analysis identified the dried sediments as having as major constituent silver, gold, and silver and gold, respectively (Fig. 2).

**Figure 3:**
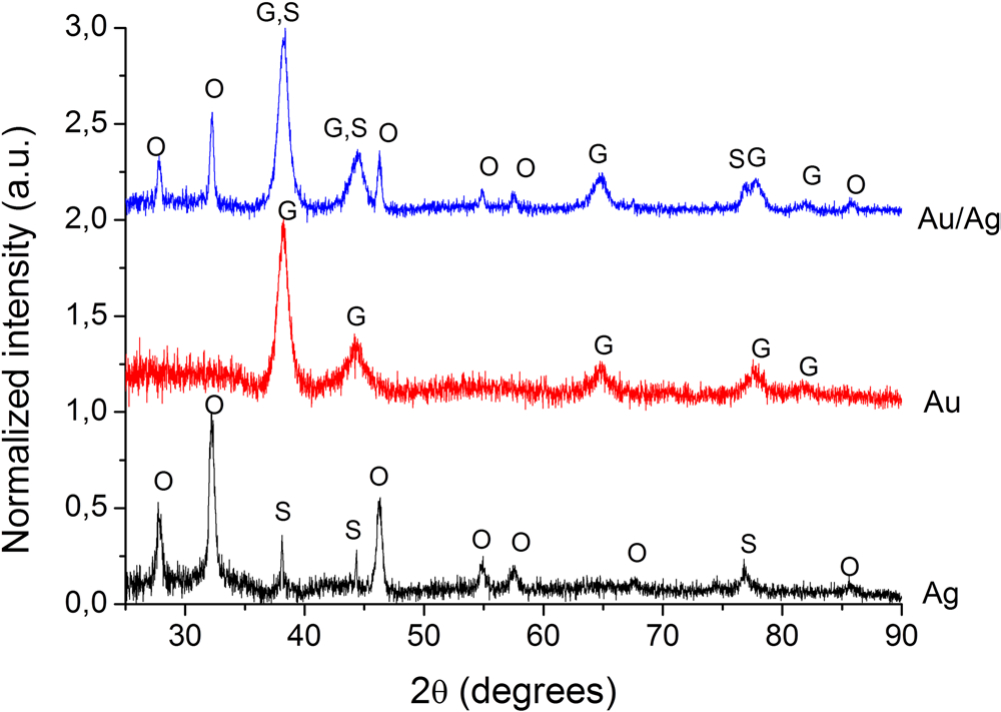
X-Ray diffractograms of the obtained nanoparticles (S – silver, O – silver oxides, G – gold).

The XRD results (Fig. 3) confirm the synthesis of the nanoparticles. In the diffractograms could be discerned the specific peaks of: silver (S, PDF card no. 01-071-4613) at 38.13, 44.31 and 77.13 degrees, corresponding to (111), (200) and, respectively, (311) planes; gold (G – 03-065-2870) at 38.16, 44.38, 64.53 and 77.75 degrees, corresponding to (111), (200), (220) and, respectively, (311) planes; for the bi-metallic nanoparticles, the peaks corresponding to the nanoparticles are found at 38.31, 44.45, 64.75, 77.01, 77.68 and 81.71 degrees. In the silver and gold/silver nanoparticles, peaks specific to silver oxides (Ag_2_O – 01-078-5867 and Ag_3_O_4_ – 03-065-9750) can also be observed, due to the oxidation of silver nanoparticles during sample preparation.

**Figure 4:**
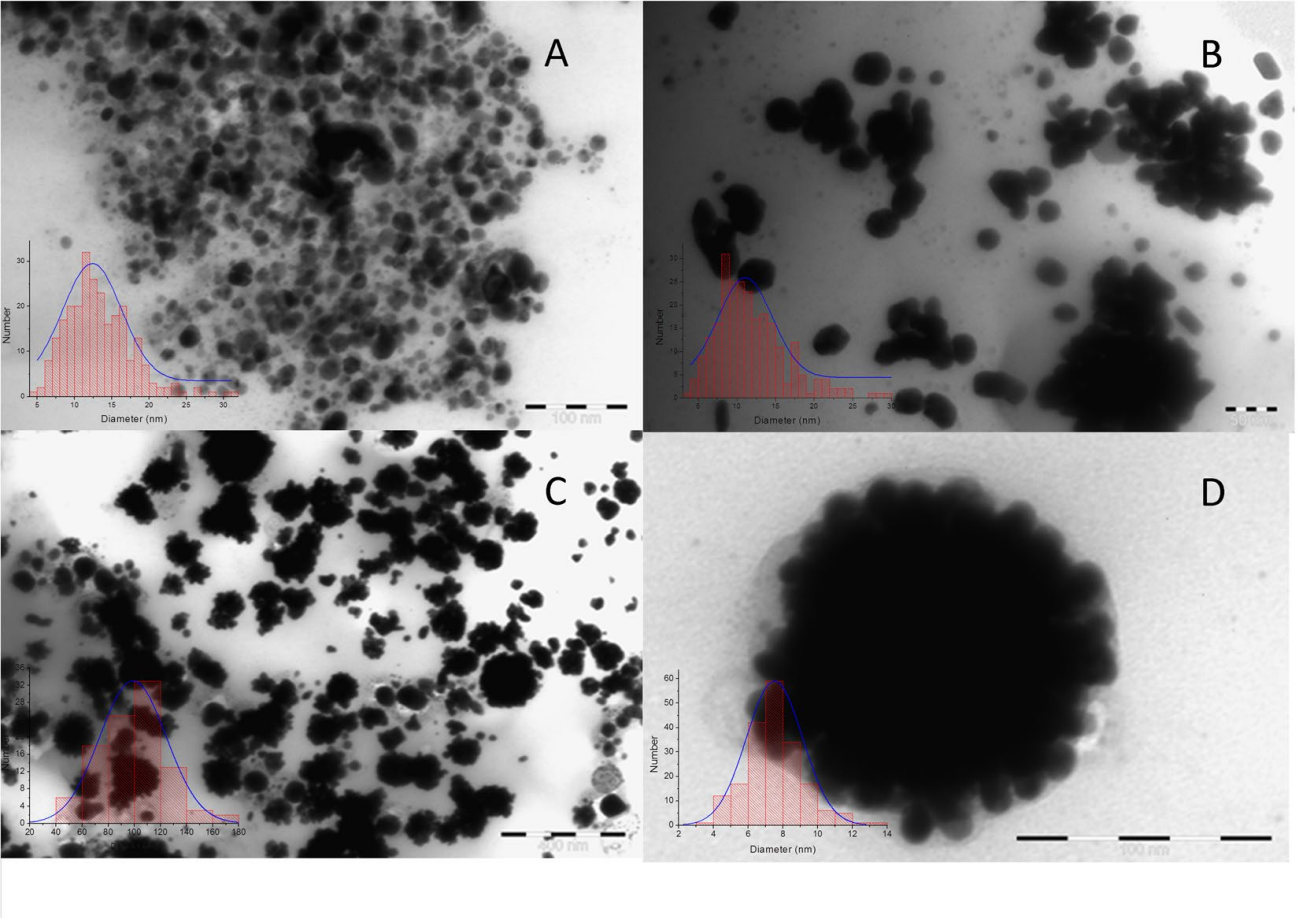
Transmission electron microscopy images and size distribution of the obtained nanoparticles (**A** – silver nanoparticles, **B** – gold nanoparticles, **C** and **D** – silver/gold nano-architectures).

Transmission electron microscopy was used to visualize the size and shape of obtained nano-architectures (Fig. 4). The dimensions of the nanoparticles (determined from over 250 measurements) had an average of 13 nm for silver nanoparticles, 10 nm for gold nanoparticles and 100 nm for the bi-metallic nano-architectures, respectively.

**Figure 5:**
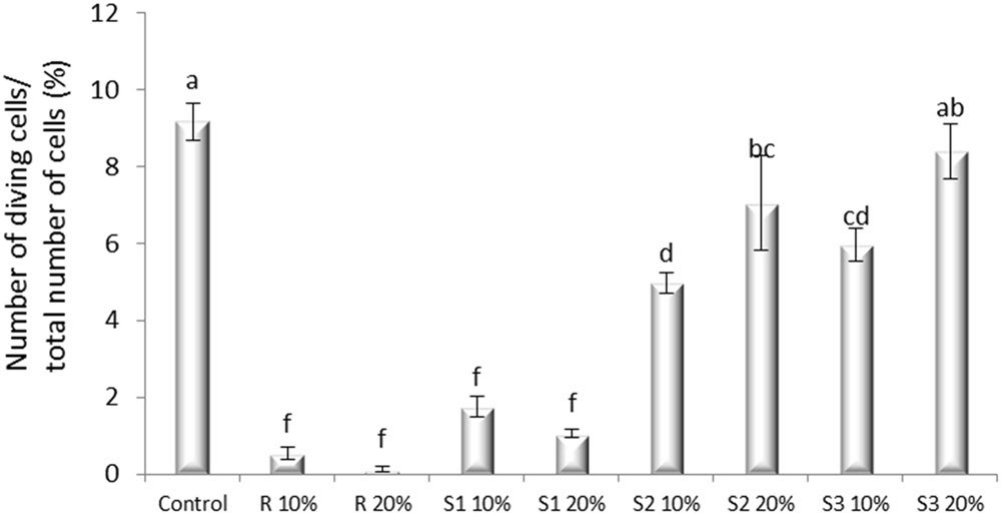
Impact *of Melissa officinalis* L. ethanolic extract and its metallic nanoarchitectures on the mitotic index of *Allium cepa* L. root cells exposed for 48 h. R – extract; S1 – silver nanoparticles solution; S2 – gold nanoparticles solution; S3 – gold/silver nanoparticles solution.

Considering the mutagenicity assay, all tested samples decreased the mitotic index when compared to the control (Fig. 5). The results demonstrated a nearly lethal effect of the extract (encoded as R). The decrease effect of R indicates a correlation with the extract concentration, since the MI of R 20% was almost null.

The phytosynthesized nanoparticles inhibited the mitodepressive effect of the *M. officinalis* extract. The activity of nanoparticles/nanoarchitectures showed a dose-response correlation, the highest concentration (20%) being more efficient to prevent the mitodepressive effect of R. The most effective in improving de *A. cepa* MI was S3 (bi-metallic nanoparticles) 20%, for which the MI was not significantly different compared to control.

Distribution of various mitotic phases showed significant dose-dependent differences from control and *R* regulated by the type of nanoparticles/nanoarchitectures. It has been noticed therefore that S2 (gold nanoparticles) 20% and S3 10% induced a significant accumulation of cells in the prophase compared to other samples (Supplementary Figure S3). This increased proportion of cells blocked in prophase may be attributed to inhibition of the formation or functioning of the spindle fibers or extension of cell cycle G_2_ stage where DNA repair occurs.

Table 3 indicates the results of mutagenicity test obtained by the *A. cepa* bioassay. Various kinds of aberrations induced by the extracts/nanoparticles are shown in Supplementary Figure S7. The most frequent clastogenic aberrations were C-mitosis, ana-telophase bridges, alongside with a low frequency of micronuclei, vagrants and laggards. Multipolarity and stickiness were very rarely noticed. Bridges and stickiness are due to chromatin dysfunction, and micronuclei, C-mitosis and laggards are due to spindle failure^33^. It worth being noticed here that different samples described by ethanolic extract *M. officinalis* with or without nanoparticles and/or nanoarchitectures induced specific chromosomal aberrations, with a characteristic and significant preponderance.

**Table 3:** Effect *of Melissa officinalis* L. ethanolic extract and its biogenic nanoparticles on chromosomal aberrations in *A. cepa* L. root meristems.

Sample exposure	Nuclear abnormalities	Chromosomes aberrations			
	Micronuclei	C-mitosis	Bridges	Vagrants	Laggards
Control	—	—	—	7.63 ± 3.82^bc^	7.67 ± 2.55^bc^
R 10%	0.13 ± 0.04^c^	—	—	0.16 ± 0.03^c^	—
R 20%	0.45 ± 0.07^c^	—	—	0.74 ± 0.08^c^	—
S1 10%	0.32 ± 0.08^c^	—	—	0.45 ± 0.06^c^	—
S1 20%	0.21 ± 0.03^c^	—	—	1.14 ± 0.13^c^	—
S2 10%	—	8.91 ± 2.5^bc^	31.06 ± 9.67^ab^	0.93 ± 00.22^c^	—
S2 20%	—	16.20 ± 8.68^bc^	48.41 ± 26.02^a^	0.74 ± 0.23^c^	—
S3 10%	—	49.73 ± 10.14^a^	16.67 ± 16.67^bc^	1.68 ± 0.20^c^	—
S3 20%	—	10.57 ± 4.15^bc^	26.11 ± 3.89^abc^	0.82 ± 0.22^c^	—

Where: R – extract, S1 – AgNP, S2-AuNP, S3 – Ag/AuNP; Values presented are mean ± SE. Means followed by the same letter not differ significantly (Duncan test, p > 0.05).

The results of the antimicrobial qualitative screening (Table 4, images presented in Supplementary Figures S8 to S13) revealed that silver nanoparticles are active against most of the tested strains (except for *Candida glabrata, Aspergillus niger, Trichoderma viride*). For *Bacillus cereus*, not only the silver nanoparticles showed a strong effect, but also the extract itself. Gold nanoparticles exhibited activity against a limited number of strains (*B. cereus –* most probably due to the extract effect*, Pseudomonas aeruginosa, C. krusei*). The gold/silver nanoparticles usually exhibit an intermediary effect (except for *Staphylococcus aureus*), similar with the values obtained for silver nanoparticles.

**Table 4:** Qualitative evaluation of antimicrobial effects of tested samples expressed by inhibition zone diameters.

No	Microbial strains	Inhibition zone diameters (mm)				
		AgNP	AuNP	Ag/AuNP	Extract	Control
1	*Staphylococcus aureus* ATCC 25923	12	0	13	0	0
2	*Bacillus cereus* B1079	20	11	17	10	0
3	*Pseudomonas aeruginosa* ATCC 27853	12	12	0	0	0
4	*Escherichia coli* ATCC 25922	15	0	12	0	0
5	*Candida. albicans* ATCC 10231	11	0	9	0	0
6	*Candida parapsilosis* ATCC 22109	10	0	10	0	0
7	*Candida glabrata* ATCC 64677	0	0	0	0	0
8	*Candida krusei* ATCC 14243	7	7	7	0	0
9	*Aspergillus niger*	0	0	0	0	0
10	*Trichoderma viride*	0	0	0	0	0

It can also be observed that the combination between silver and gold (in the form of core-shell architectures) decreased the antimicrobial effect of silver nanoparticles, expressed by smaller inhibition zone diameters and higher MIC values (Tables 4 and 5). The results of the quantitative assay (determination of the minimum inhibitory concentration) are presented in Table 5, while their graphic representation is presented in Supplementary Figures S14 to S21.

**Table 5:** Minimum inhibitory concentration of tested samples.

No.	Microbial strains	Minimal inhibitory concentration values			
		AgNP (mM)	AuNP (mM)	Ag/AuNP (mM)	Extract
1	*Staphylococcus aureus* ATCC 25923	0.0125	0.1	0.025	Undiluted
2	*Bacillus cereus* B1079	0.0015625	0.2	0.003125	Undiluted
3	*Pseudomonas aeruginosa* ATCC 27853	0.003125	0.2	0.0125	Undiluted
4	*Escherichia coli* ATCC 25922	0.00625	0.2	0.0125	Undiluted
5	*Candida. albicans* ATCC 10231	0.003125	0.2	0.00625	Undiluted
6	*Candida parapsilosis* ATCC 22109	0.0125	0.025	0.025	Undiluted
7	*Candida glabrata* ATCC 64677	0.05	0.2	0.1	Undiluted
8	*Candida krusei* ATCC 14243	0.05	0.4	0.1	Undiluted
9	*Aspergillus niger*	0.8	0.8	0.8	Undiluted
10	*Trichoderma viride*	0.05	0.8	0.05	Undiluted

The results of the evaluation of biofilm eradication properties are presented in Table 6, while their graphic representations are shown in Supplementary Figures S22 to S28.

**Table 6:** Minimal concentration for biofilm eradication values for tested samples, expressed in mM and FD – dilution factor (for the extract).

No.	Microbial strains	Minimal concentration for biofilm eradication				
		AgNP (mM)	AuNP (mM)	Ag/AuNP (mM)	Extract (dilution)	Control (mM)
1	*Staphylococcus aureus* ATCC 25923	0.0125	0.4	0.025	FD2	1712
2	*Bacillus cereus* B1079	0.0015625	0.2	0.003125	FD4	856
3	*Pseudomonas aeruginosa* ATCC 27853	0.00625	0.2	0.00625	FD4	856
4	*Escherichia coli* ATCC 25922	0.0125	0.4	0.0125	FD2	856
5	*Candida. albicans* ATCC 10231	0.00625	0.2	0.00625	FD4	856
6	*Candida parapsilosis* ATCC 22109	0.025	0.2	0.05	FD4	428
7	*Candida glabrata* ATCC 64677	0.00625	0.2	0.00625	FD4	856
8	*Candida krusei* ATCC 14243	0.05	0.4	0.1	FD4	856

## Discussions

The synthesis of noble metal nanoparticles using either microorganisms or plant extracts has emerged in the last decades as an alternative approach to the classical (either physical or chemical) methods, due to their several advantages: they are simple (usually one-pot), cost-effective, give high yields, yet non-toxic and therefore environmentally friendly^34^. Although it has the advantages of green chemistry approach, the use of microorganisms (such as bacteria, yeasts, actinomycetes or fungi) has certain limitations, such as the difficulty of implementing on a large scale and the need for maintaining cell cultures. Thus, the use of plant extracts is distinguished by other methods in that the method is simple and scalable^35^.

As a first step of the analytic protocol developed for the current study, the extract was subjected to chromatographic analyses. By means of the GC-MS analysis performed, 67 peaks were identified using the databases, from a total of 75 peaks found to be present in the extract. The area of the 8 unidentified peaks totalizes ca. 3.33%.

Following the identification of the volatile components, the HPLC analyses were performed in order to quantify different compounds as markers of *M. officinalis* extract^36–38^.

The obtained HPLC results are consistent with the literature data regarding *M. officinalis* extracts. For example, Arceusz and Wesolowski^36^, in a study covering 19 lemon balm samples from 12 Polish manufacturers, achieved values ranging from 0.15 to 42.3 mg/g for rosmarinic acid, from 0.006 to 1.59 mg/g for ferulic acid, from 0.01 to 0.33 mg/g for chlorogenic acid and from under the detection limit to 0.067 mg/g for gallic acid; Arceusz *et al*.^39^ identified rutin (from under the detection limit to 2.34 mg/g) and quercetin (0.17–27 mg/g) in different types of extract obtained from lemon balm.

Upon the addition of the reducing agent (extract) a color change can be rapidly observed. The change of solution color is an indicator of the nanoparticles formation: a yellowish color is specific for small size silver nanoparticles; a ruby-red color is specific to gold nanoparticles, while the bi-metallic nanoparticles are characterized by a dark brownish color^40^.

The UV-Vis spectra present characteristic absorption peak for silver nanoparticles (Fig. 1A) at 417 nm (suggesting particle dimensions under 30 nm) and gold nanoparticles (Fig. 1B) at 537 nm (suggesting particle dimensions around 50 nm). The spectrum in Fig. 1C presents a decrease of the specific peak of gold nanoparticles (accompanied by a slight shift, to 541 nm) and an increase of the silver peak (with a slight shift to 413 nm). The peaks variation obtained for silver and gold plasmon bands suggest core-shell architecture, with a coating of silver around the gold nanoparticles^37^.

**Figure 6:**
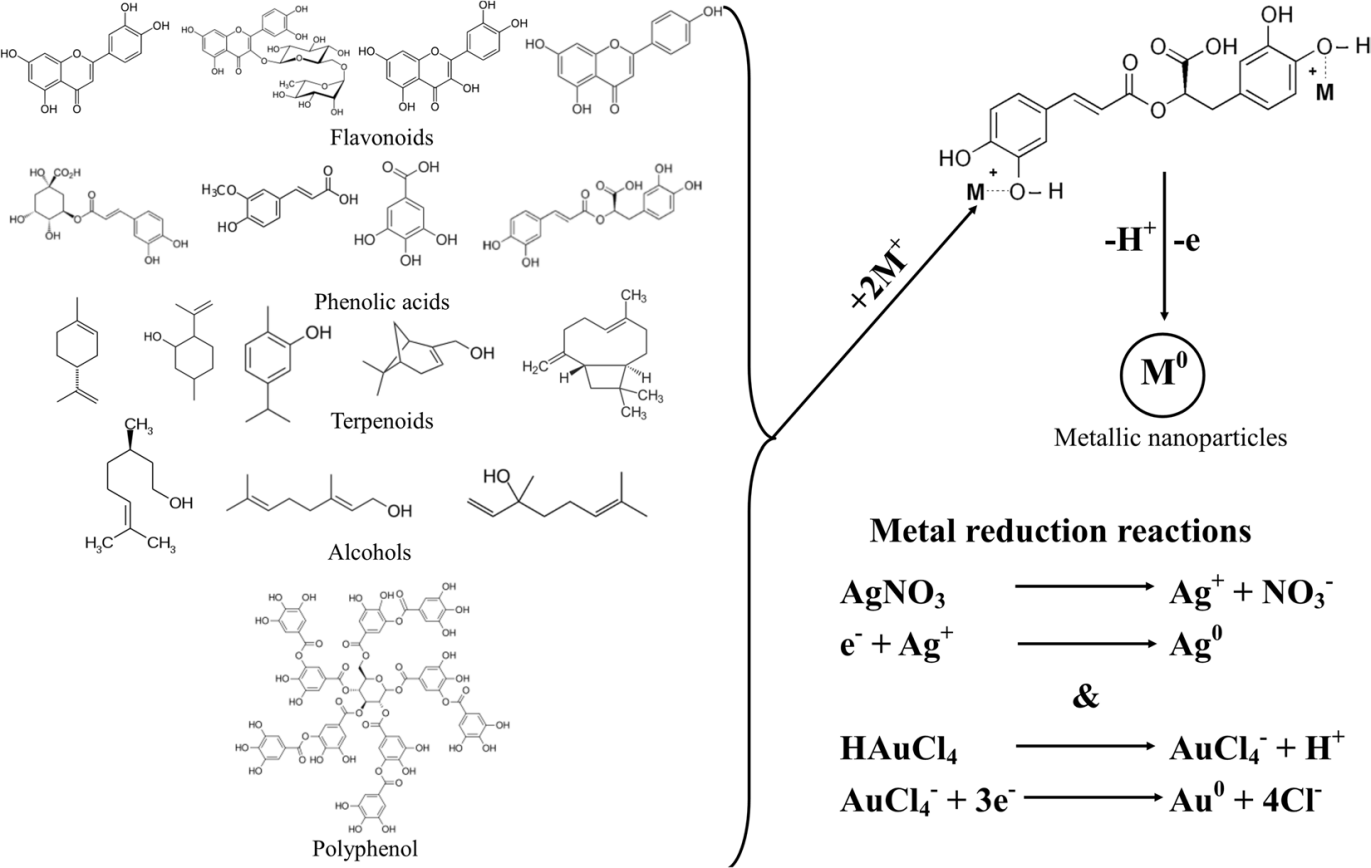
Proposed mechanism for the synthesis of metal nanoparticles using plant extracts (adapted from literature data^3,41,62^) and the reactions involved in the metal reduction to nanoparticle.

The exact pathway involved in the reduction of metallic ions to metallic nanoparticles is still under debate, several mechanisms being proposed by different authors focusing on different active compounds, including polyphenols^41^, flavonoids^42^ and other bio-active compounds^43^. Figure 6 presents both the proposed mechanism and the involved chemical reactions for metal reduction using plant extracts.

In our opinion, the assignment of a particular active substance as the main reducing agent is hazardous, as, most probably, the combination of various biomolecules found in natural extracts acts both as a reducing and stabilizing agent, in a synergic manner^43^. Moreover, we consider that the most probable biomolecules involved in the reduction of metallic salts to nanoparticles are the phenolic compounds, as several authors proposed^35,41^.

Different studies stressed out that plant metabolites like polyphenols are natural reducing agents^41^ and capping agent with contribution to metallic nanoparticles stabilization. This effect was most emphasized when different solvent extraction was used and the quantities of polyphenols had significant variation, hence the effectiveness in the formation and stabilization of nanoparticles also varied^44^. In present study, the HPLC analysis was carried out in order to link the formation of NPs to some of the metabolites found in *Melissa officinalis* L. extract. Besides usual metabolites (gallic acid, quercetin, rutin, etc.) which can be responsible for NPs synthesis^45^ it was found that rosmarinic acid is present in large amount in studied extract. This compound is very effective in NPs synthesis according to literature data^46^. It was found that the rosmarinic acid molecules are able to generate and stabilize the gold and silver nanoparticles. Rutin was also found in large quantities in plant extract and therefore can act as a reducing and capping agent in the process.

The GC-MS analyses, complementary to the HPLC determinations, identified some metabolites (numbers 13, 15, 38, 44, 48, 57, 62, 65, 66 and 75 from Table 1) in the *Melissa officinalis* L. extract, known to present antifouling capacity, which could contribute to stabilization of the nanoparticles^47^.

The XRD analysis, besides confirmation of the phytosynthesis of the nanoparticles, also provides information regarding the particle dimensions: the crystallite size, determined by the Debye-Scherrer equation, showed silver nanoparticles with an average dimension of 14 nm and gold nanoparticles with an average dimension of 10 nm. Similar dimensions were obtained for the bi-metallic nanoparticles (ca. 8.5 nm).

The investigation of the samples using transmission electron microscopy (as presented in Fig. 4) confirmed the findings of XRD. Uniform silver nanoparticles could be observed, with an average dimension around 13 nm (determined from over 250 measurements), in a relatively narrow range of dimensions (Fig. 4A). The analysis of gold nanoparticles revealed smaller nanoparticles (around 10 nm, determined from over 250 measurements), but very inhomogeneous in shape (Fig. 4B). Besides spherical nanoparticles different shaped particles, such as triangular, hexagonal, rhombic, etc. could be observed. A very interesting case is observed for the bi-metallic nanoparticles: at a first glance (Fig. 4C), it appears that very big particles were formed, with dimensions around 100 nm, with some dispersed smaller particles. A closer look (Fig. 4D) reveals that the large particles are actually clusters of very small nanoparticles (around 8 nm), that tend to aggregate in a flower-like core-shell configuration, as other authors previously reported^48,49^.

The *Allium cepa* test system is widely used as an ideal bioindicator for the first screening of genotoxicity, as a suitable indicator for the evaluation of clastogenic and/or aneugenic effects of a potential genotoxic agent^50^, providing similar results with *in vitro* animal test systems^51^.

The reduction of the mitotic index was usually associated with a significant increase in the frequency of cells in metaphase, when comparing with control (Supplementary Figure S4). Samples defined by the biogenic Ag and Au nanoparticles at a concentration of 10% proved to be valuable statmokinetic agent. Significant incidence of metaphase in the root tips of *A. cepa* L. incubated with S3 10%, suggested a lower mitostimulatory effect of these nanoarchitectures.

An increased number of anaphase associated to a reduced number of telophase was observed for the slides defined by ethanolic extract of *M. officinalis*, irrespective of the tested concentration. However, statistical analysis revealed no significant differences between the samples S1, S2, S3 for their effect on the rate of anaphase and telophase (Supplementary Figures S5 and S6).

Formation of micronuclei in a significant low frequency was characteristic for those meristematic root cells of *A. cepa* incubated in R and S1, suggesting a minor toxicity of R along with a lack of chemoprotective activity of Ag nanoparticles. According to Carvalho *et al*.^52^ ethanolic and aqueous extracts of *M. officinalis* induced a low frequency of micronucleated cells in CF-1 male mice, but not significant enough to indicate mutagenic and genotoxic activities. Moreover, the study of Kamdem *et al*.^53^ did not reveal either the cytotoxic nor genotoxic effects of *M. officinalis* extract to human leukocytes. In this study, the formation of micronuclei might be due to either the 48 hours contact of meristematic root tips with ethanol used as solvent for extract preparation or to inappropriate concentrations.

Microscopic analysis revealed a protective effect of *M. officinalis* extracts containing Au nanoparticles or Ag/Au nano-architectures on nuclear DNA damage, expressed in absence of micronuclei. Meristematic root cells of *A. cepa* exposed to S2 and S3 for 48 h were affected by turbagenic changes, such as C-mitosis and bridges. Significant high frequency of C-mitosis observed in root tip cells incubated in particular with S3 10%, recommend Ag/Au nano-architecture as a potential antitumor agent.

From the presented antimicrobial assays, very small MIC values are noticed for silver nanoparticles solution obtained for most of the tested strains (except *Aspergillus niger* strain). *A. niger* is known to be the most resistant pathogen among fungi. The resistance of this strain to AgNPs may result either from the activity of some chemical components which are produced by *A. niger* and inhibits the bioactivity mechanisms of the silver ions or from the structure particularity of the cell wall^54^. Nevertheless, details about the specific resistance mechanism need to be further investigated.

The antimicrobial activity of silver nanoparticles has been reported against fungus, yeast, Gram-negative and Gram-positive bacteria^55,56^. Silver nanoparticles (AgNP) are known to possess antimicrobial properties, but the mechanisms regarding their microbial toxicity are not fully known. One of the hypotheses is that silver nanoparticles can cause cell lysis or inhibit cell growth, thereby causing structural changes in the cell membrane like the permeability of the cell membrane and consequently death of the cell^56,57^. The results of Hsueh *et al*.^58^ confirmed that AgNP toxicity is likely mediated by the released Ag^+^ ions from AgNPs, which penetrate bacterial cells and are subsequently intracellularly oxidized to Ag_2_O. These findings provide conclusive evidence for the role of Ag^+^ ions in AgNP microbial toxicity^58^.

When comparing our results with literature data, it should be noticed that the MIC values are better even than the ones obtained for silver nanoparticles obtained *via* chemical route: Kim^59^ *et al*. obtained MIC values of 0.033 mM and 0.0033 mM against *S. aureus* and, respectively, *E. coli*, while Guzman *et al*.^60^ obtained MIC values ranging from 14.38 to 259 mg/L (for *S. aureus* and *E. coli*) and from 6.74 to 215.74 mg/L for *P. aeruginosa*, using silver nanoparticles synthesized *via* citrate method. However, when comparing results regarding antimicrobial properties of phytosynthesized nanomaterials with the properties of nanoparticles obtained using classical chemical or physical methods, we must consider that “green” nanoparticles have the advantage of a functionalized surface due to presence of bioactive molecules, such as organic ligands, proteins, polysaccharides, and polyatomic alcohols^61^.

Regarding the assessment of adherence on the inert substratum assay, it is worth to notice that the silver nanoparticles appeared active against all studied strains, while the gold nanoparticles showed no effect on the adherence on inert substratum. The bi-metallic nanoparticles are active against all studied strains, with MCBE (minimal concentration for biofilm eradication) values higher than the ones obtained for silver nanoparticles.

In natural sites, microorganisms can adhere to biotic or abiotic surfaces, generating biofilms^62^. Because of the medical and industrial implication, this phenomenon has been studied in many different environments, generating new control strategies that follow the use of different anti-biofilm strategies like bio-solutions (enzymes, antimicrobial peptides, quorum sensing molecules, plant extracts) or nanoparticles^63,64^. In their research, Kalishwaralal *et al*.^65^ treated *Pseudomonas aeruginosa* and *Staphylococcus epidermidis* with silver nanoparticles over 24 h, obtaining more than 95% inhibition in biofilm formation. Also, another recent study showed that AgNPs are also effective against *Mycobacterium spp*. biofilms^66^. Silver nanoparticles express synergistic activity with ampicillin, kanamycin, streptomycin or vancomycin against *E. coli* and *P. Aeruginosa* ^67^. Other studies have revealed synergy of AgNPs with compounds other than antibiotics. As an example, Ammons *et al*.^68^ showed that a silver wound dressing combined with the immune molecule lactoferrin and the rare sugar-alcohol xylitol, reduced biofilm viability more effectively than standard silver hydrogel. Our results are in agreement with previous studies, the phytosynthesized nanoparticles inhibiting the adherence capacity of the majority of tested strains, the MCBE values being very low (under 0.05 mM).

## Conclusions

By utilizing an eco-friendly route, three types of nanoparticles were obtained, *i.e*. silver and gold nanoparticles, and silver/gold nano-architectures. The analytical results proved the successful phytosynthesis, offering information regarding their size and morphology.

The obtained nanomaterials were tested for their mutagenicity and antimicrobial properties. Mitosis was inhibited by ethanolic extract of *M. officinalis*. The Ag nanoparticles were not active on nuclear DNA damage. The Au nanoparticles appeared nucleoprotective, but were aggressive in generating clastogenic aberrations in *A. cepa* root meristematic cells.

The evaluation of the antimicrobial properties of the phytosynthesized nanoparticles showed that silver nanoparticles are active against most of the tested strains (especially for adherence on inert substratum). From both assays, it can be concluded that the bi-metallic nanoparticles seem to be the most promising materials for further applications.

## Electronic supplementary material

Supplementary material
