# Supplementary material for: Phyto-mediated metallic nano-architectures via Melissa officinalis L.: synthesis, characterization and biological properties
Source: Sci Rep. 2017 Sep 29;7:12428. doi: 10.1038/s41598-017-12804-7 (PMC5622205; doi:10.1038/s41598-017-12804-7)
Supplement: Supplementary file 1 — Supplementary material [file 41598_2017_12804_MOESM1_ESM.pdf]

SUPPLEMENTARY MATERIAL

Phyto-mediated metallic nano-architectures *via Melissa officinalis* L.: synthesis, characterization and biological properties

Irina Fierascu[1,2†], Milen I. Georgiev[1,3†], Alina Ortan[1*†], Radu Claudiu Fierascu[1,2*†], Sorin Marius Avramescu[1,4], Daniela Ionescu[1,5], Anca Sutan[6], Alexandru Brinzan[7], Lia Mara Ditu[8]

[1] *University of Agronomic Science and Veterinary Medicine, 59 Marasti Blvd, 011464 Bucharest, Romania*
[2] *The National Institute for Research & Development in Chemistry and Petrochemistry-ICECHIM, 202 Spl. Independentei, 060021 Bucharest, Romania*
[3] *Laboratory of Applied Biotechnologies, Institute of Microbiology, Bulgarian Academy of Sciences, 139 Ruski Boulevard, 4000, Plovdiv, Bulgaria*
[4] *University of Bucharest-PROTMED Research center, 36-46 M. Kogalniceanu Blvd., 050107 Bucharest, Romania*
[5] *S.C. HOFIGAL EXPORT IMPORT S.A., 2 Intrarea Serelor, 042124, sector 4, Bucharest, Romania*
[6] *University of Pitesti, Faculty of Science, 1 Targu din Vale Str., 110040 Pitesti, Romania*
[7] *Romanian Academy, Institute of Biology – Bucharest, 296 Spl. Independentei, 060031 Bucharest, Romania*
[8] *University of Bucharest, Microbiology Department, 1-3 Aleea Portocalelor, 060101, Bucharest, Romania*
**Corresponding authors: Alina Ortan, 59 Marasti Blvd, 011464, Bucharest, Romania, phone: +40771323216, e-mail: alina_ortan@hotmail.com and Radu Claudiu Fierascu, 202 Spl. Independentei, 060021, Bucharest, Romania, phone: +40723421590, e-mail: radu_claudiu_fierascu@yahoo.com*
[†] These authors had an equal contribution to the present paper and are considered main authors of this study.

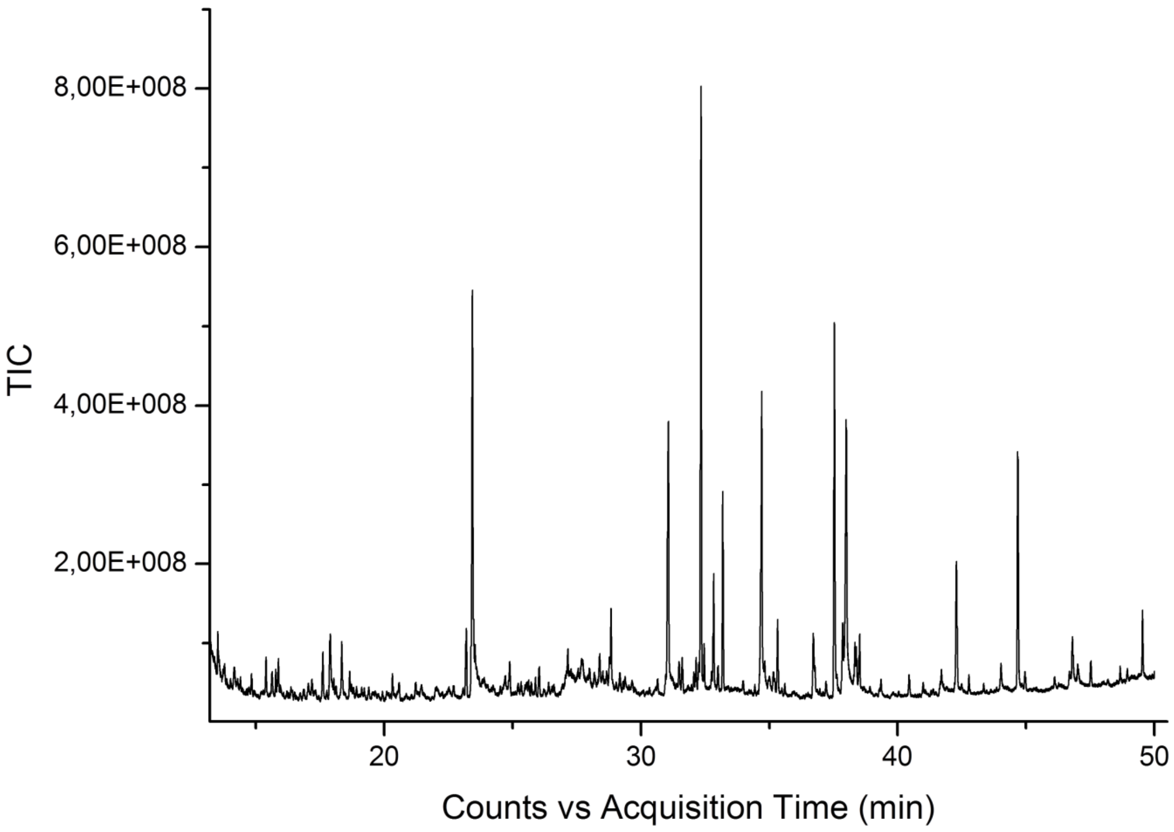

Figure S1. GC-MS chromatogram of the extract

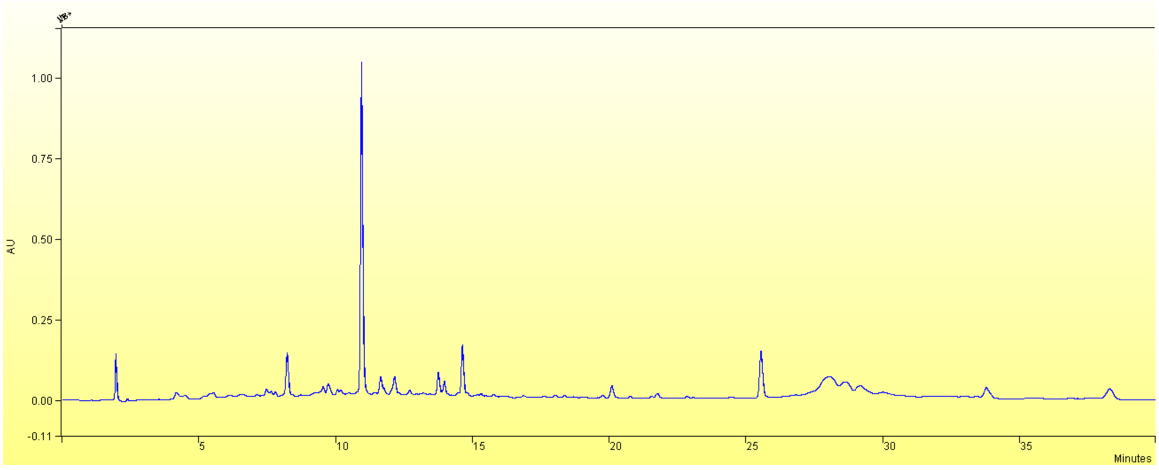

Figure S2. HPLC chromatogram of the extract

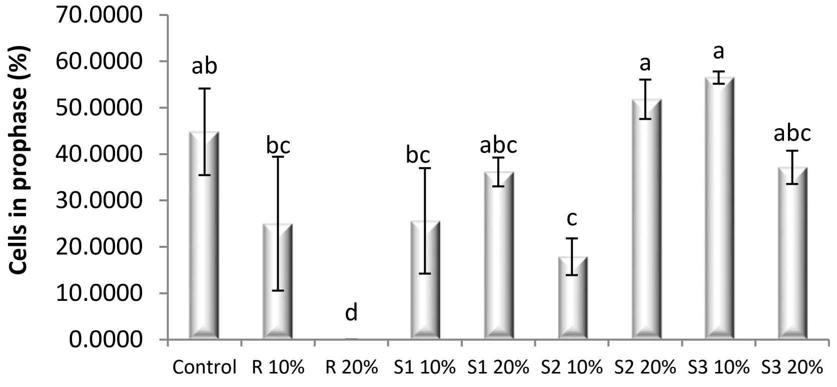

Figure S3. Effect *of Melissa officinalis* L. ethanolic extract and its biogenic nanoparticles on the prophase stage of *Allium cepa* L. root cells exposed for 48 h (a,b,c,d: the interpretation of the significance of the differences by means of the Duncan test, p<0.05).

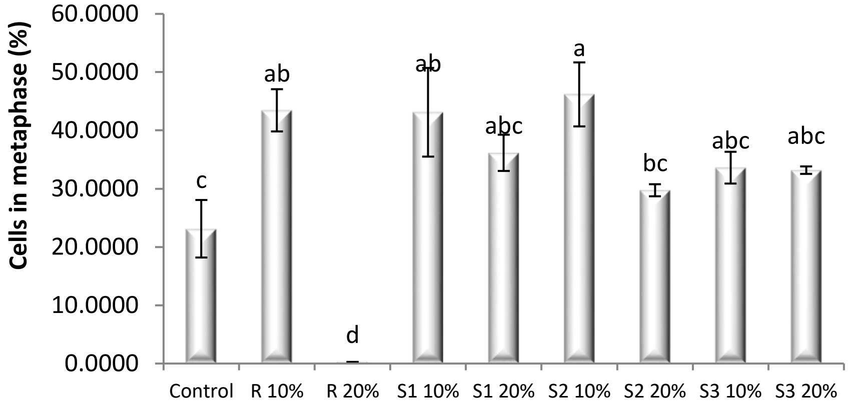

Figure S4. Effect *of Melissa officinalis* L. ethanolic extract and its biogenic nanoparticles on the metaphase stage of *Allium cepa* L. root cells exposed for 48 h (a,b,c,d: the interpretation of the significance of the differences by means of the Duncan test, p<0.05).

.

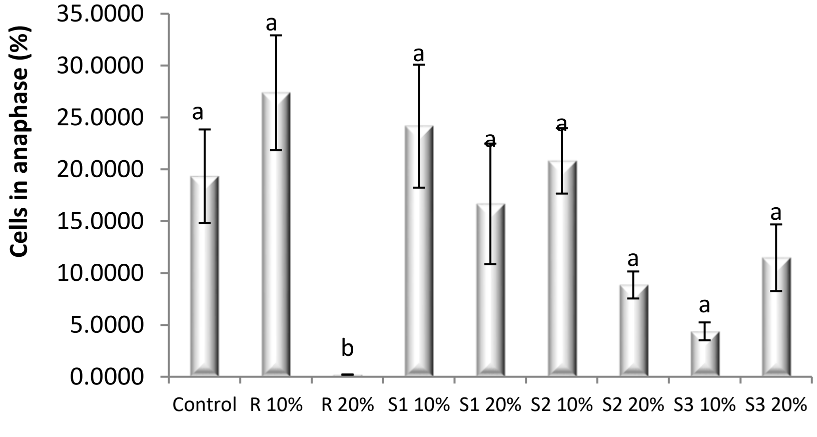

Figure S5. Effect of *Melissa officinalis* L. ethanolic extract and its biogenic nanoparticles on the anaphase stage of *Allium cepa* L. root cells exposed for 48 h (a: the interpretation of the significance of the differences by means of the Duncan test, p<0.05).

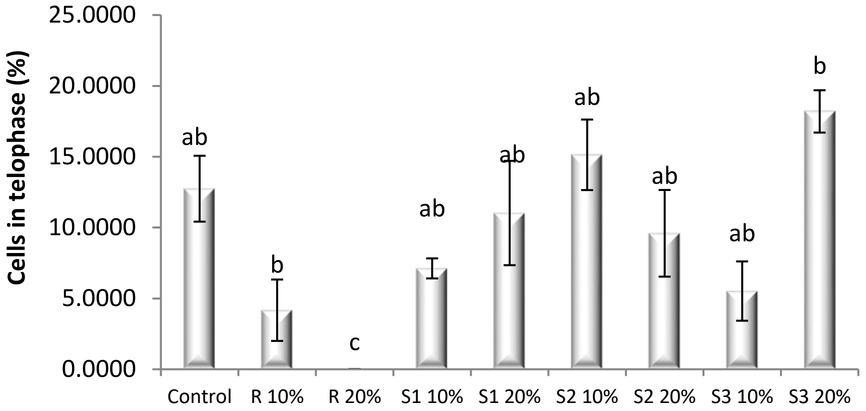

Figure S6. Effect *of Melissa officinalis* L. ethanolic extract and its biogenic nanoparticles on the telophase stage of *Allium cepa* L. root cells exposed for 48 h (a,b: the interpretation of the significance of the differences by means of the Duncan test, p<0.05).

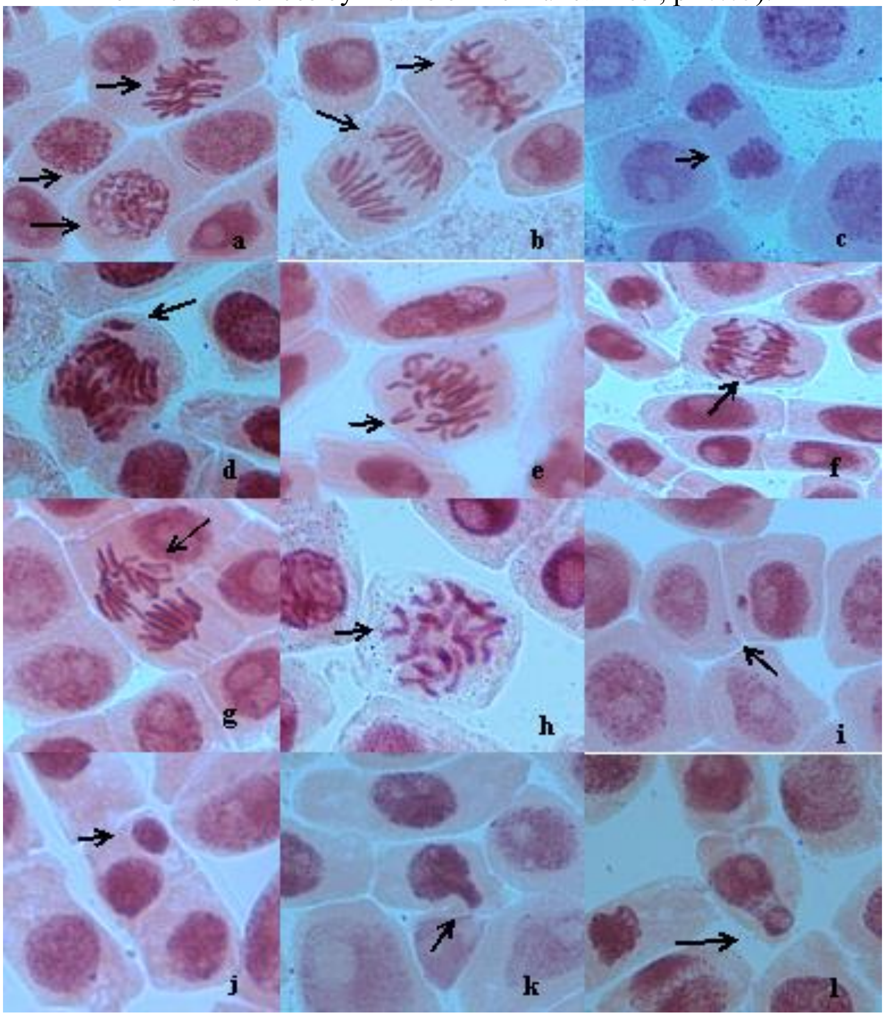

Figure S7. Chromosomal aberrations identified in root meristematic cells of *Allium cepa* L. that underwent treatment with ethanolic extracts of *Melissa officinalis* L. and phytosintesized Ag, Au nanoparticles, and Ag+Au nanoarchitectures (a, b, c) normal prophase, metaphase, anaphase, telophase - Control; (d) laggards – S2 10%; (e) vagrants – S2 10% (f) anaphase bridges – S2 20% (g) disturbed anaphases showing chromosomes bridge and laggards – S2 20%; (h) C-mitosis – S3 10%; (i, j) micronuclei - R 20%; (k,l) nuclear budding – R 10%.

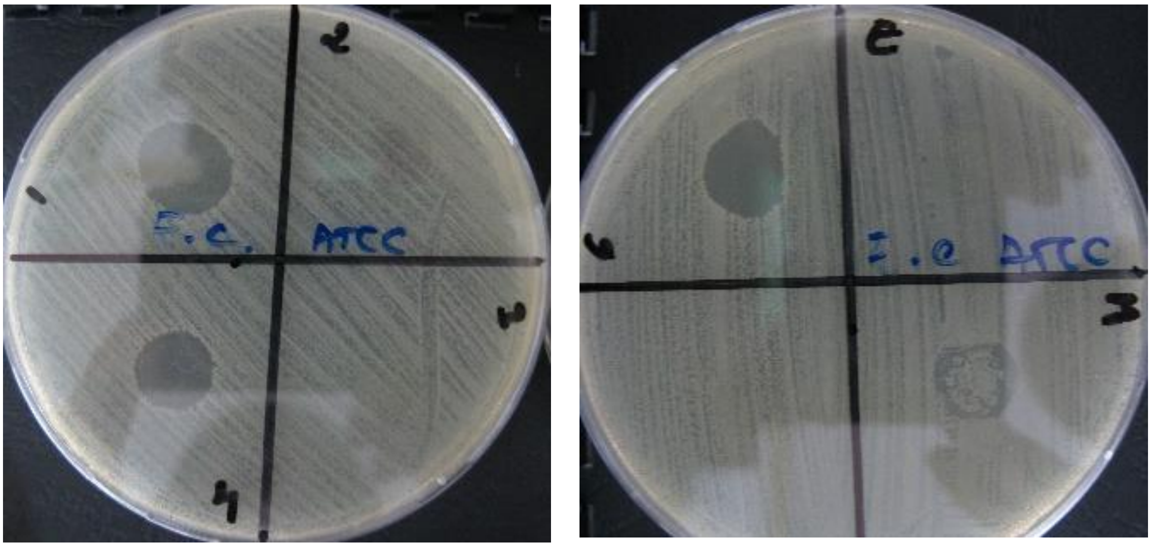

a b

Figure S8. Aspects of the inhibition zone for *Escherichia coli* ATCC 25922; 1-AgNP 0.8 mM Ag, 2-AuNP 0.8 mM Au, 4-Ag/AuNP 0.4 mM Ag/0.4 mM Au; E – extract; M - Control ethanol 96%.

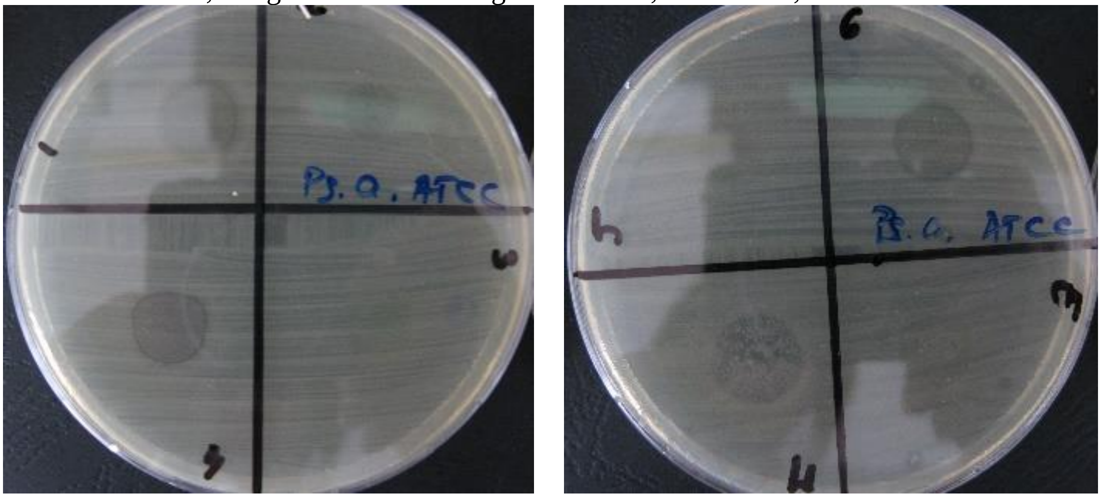

a b

Figure S9. Aspects of the inhibition zone for *Pseudomonas aeruginosa* ATCC 27853

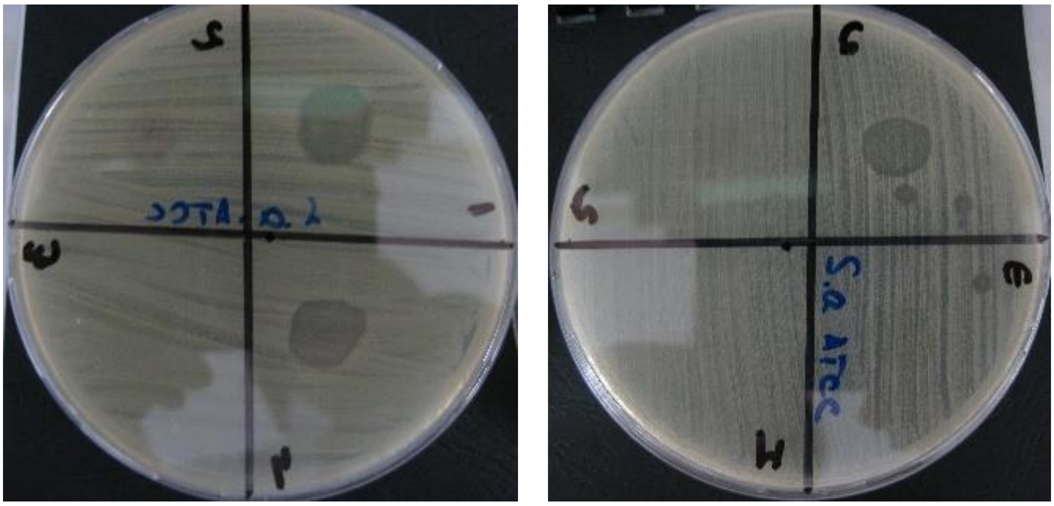

a b

Figure S10. Aspects of the inhibition zone for *Staphylococcus aureus* ATCC 25923

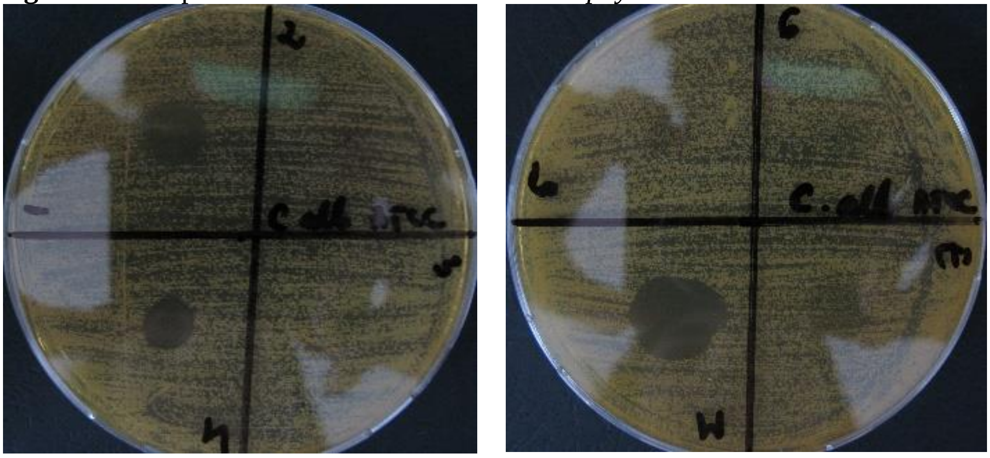

a b

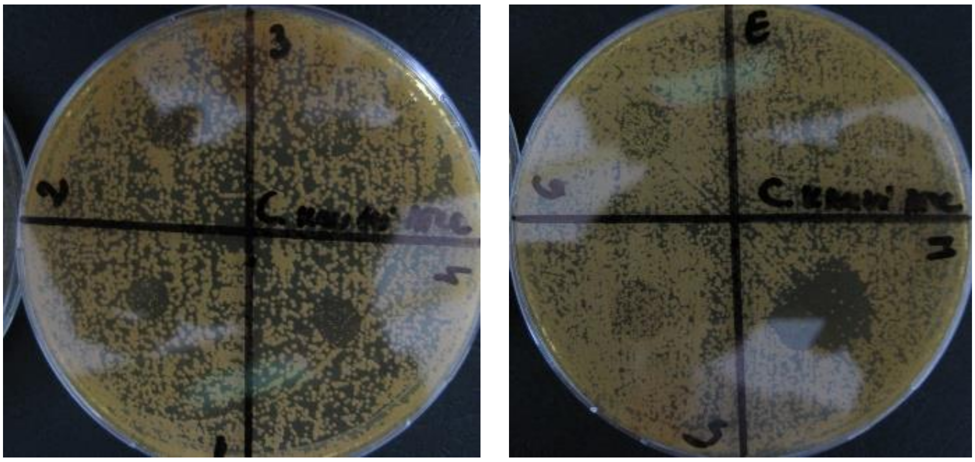

c d

Figure S11. Aspects of the inhibition zone for *Candida sp.*

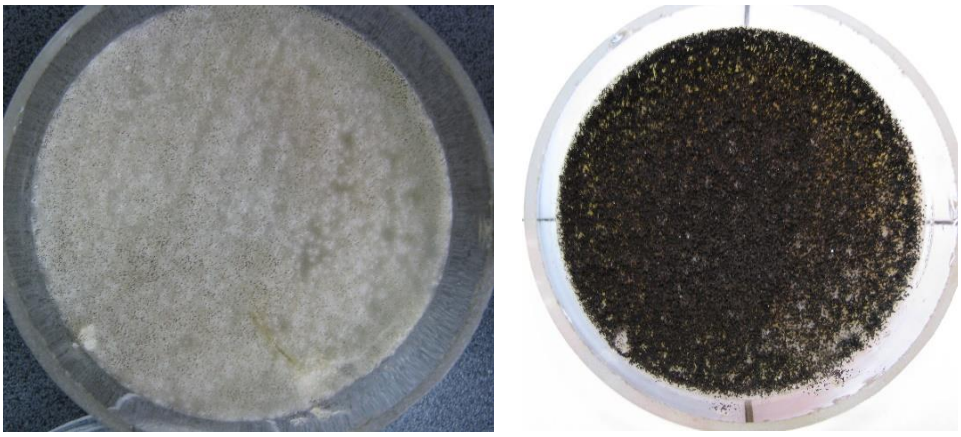

a b

Figure S12. *Aspergillus niger* culture: a. 3 days; b. 7 days – no inhibition zone after the contact with tested compounds

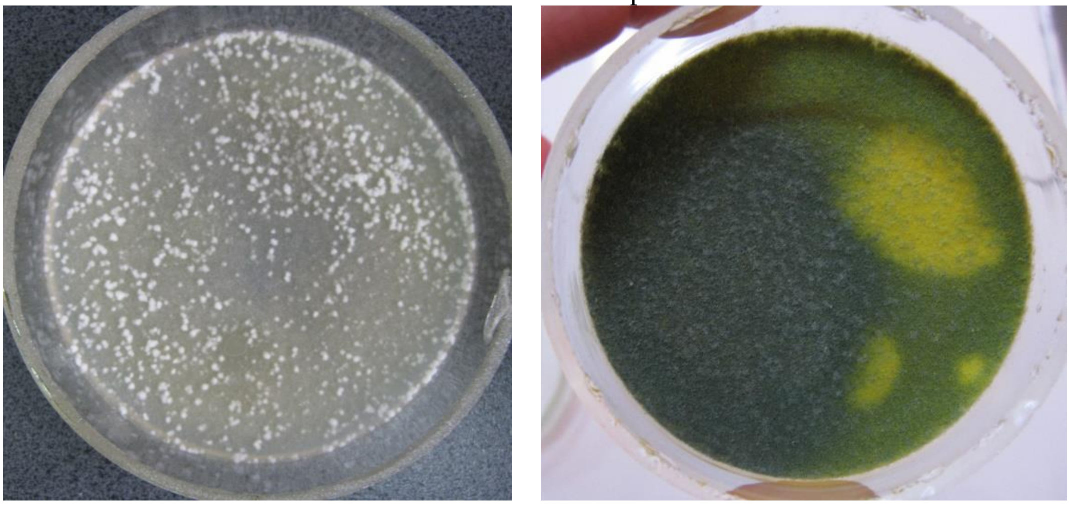

a b

Figure S13. *Trichoderma viride* culture: a. 3 days; b. 7 days - no inhibition zone after the contact with tested compounds

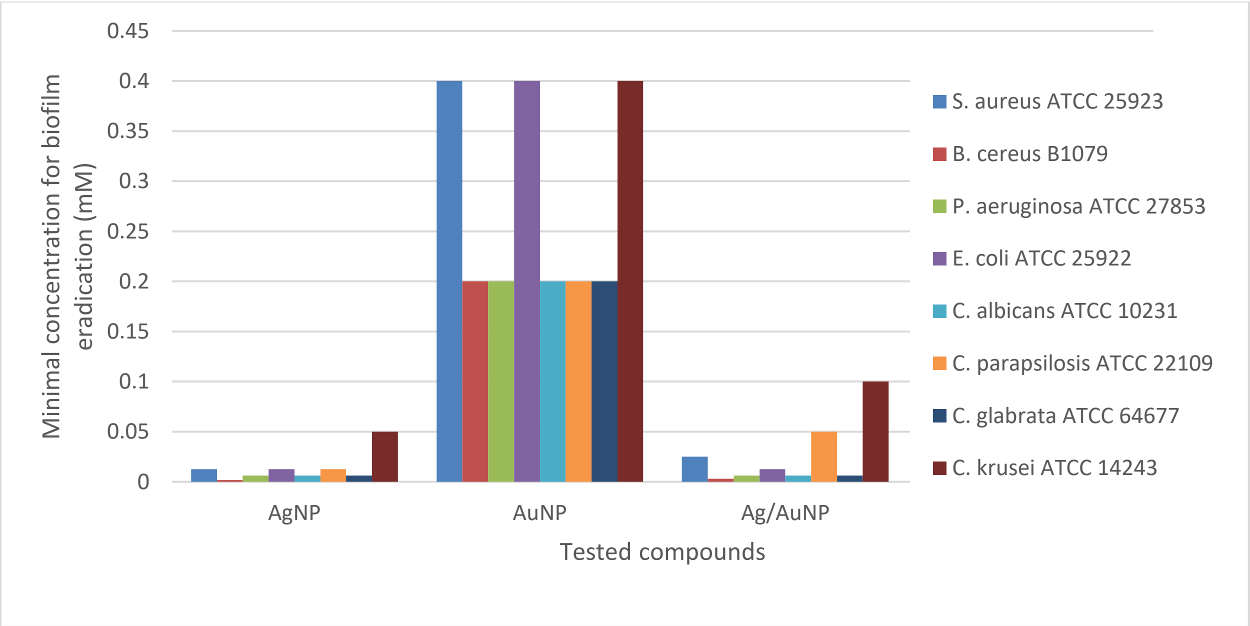

Figure S14. Graphic representation of minimal inhibitory concentration values.

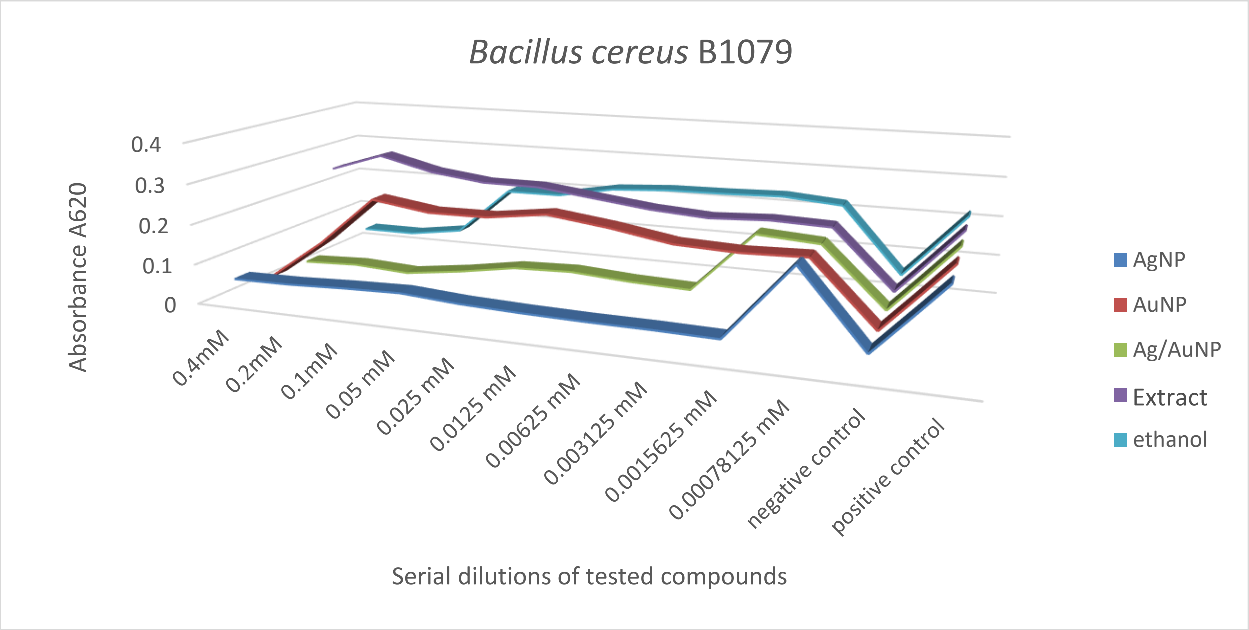

Figure S15. Graphic representation of minimal inhibitory concentration values after spectrofotometric reading of *Bacillus cereus* B1079 culture

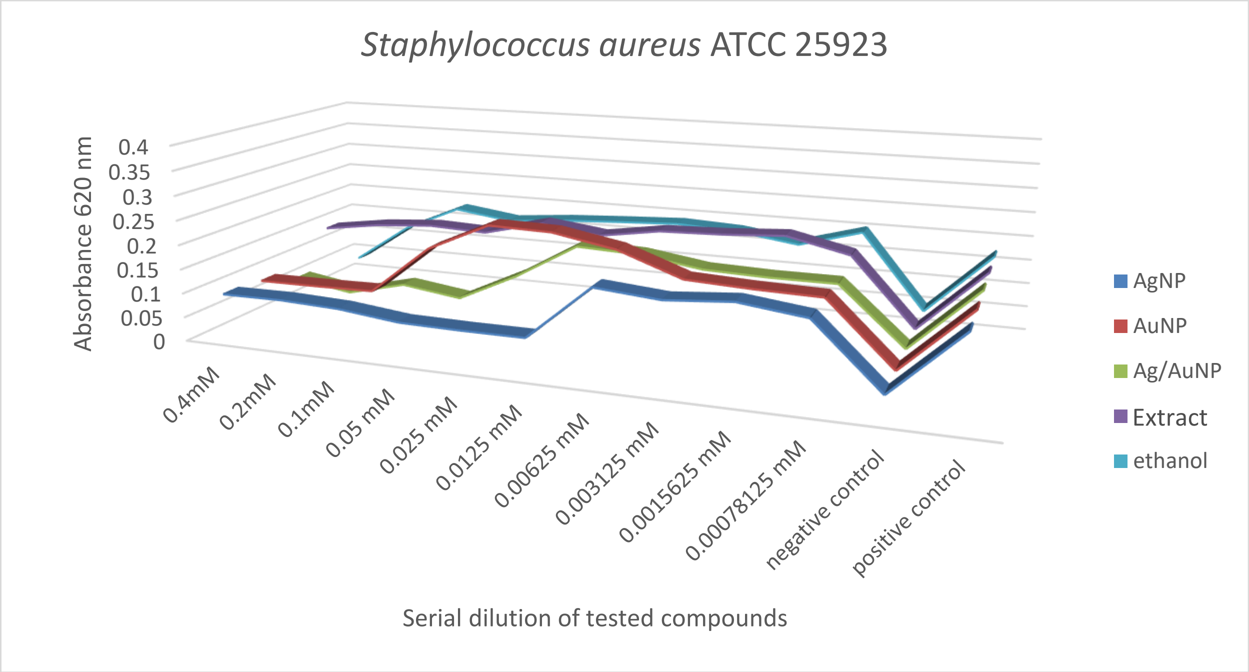

Figure S16. Graphic representation of minimal inhibitory concentration values after spectrofotometric reading of *S. aureus* ATCC 25923 culture

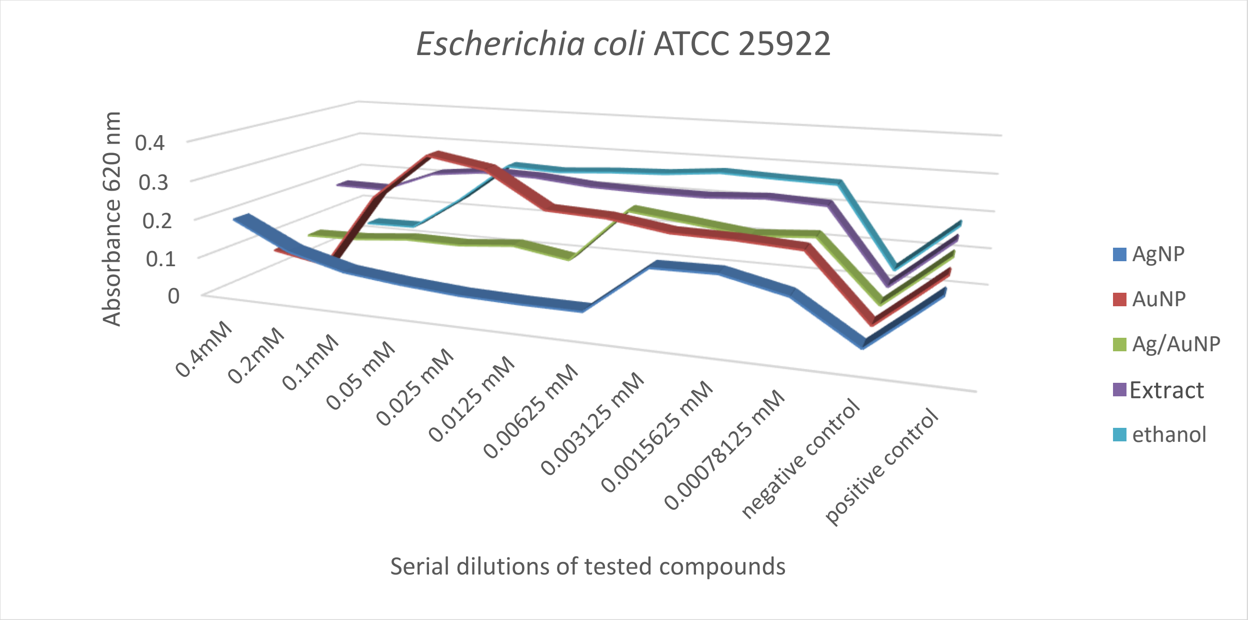

Figure S17. Graphic representation of minimal inhibitory concentration values after spectrofotometric reading of *E coli* ATCC 25922 culture

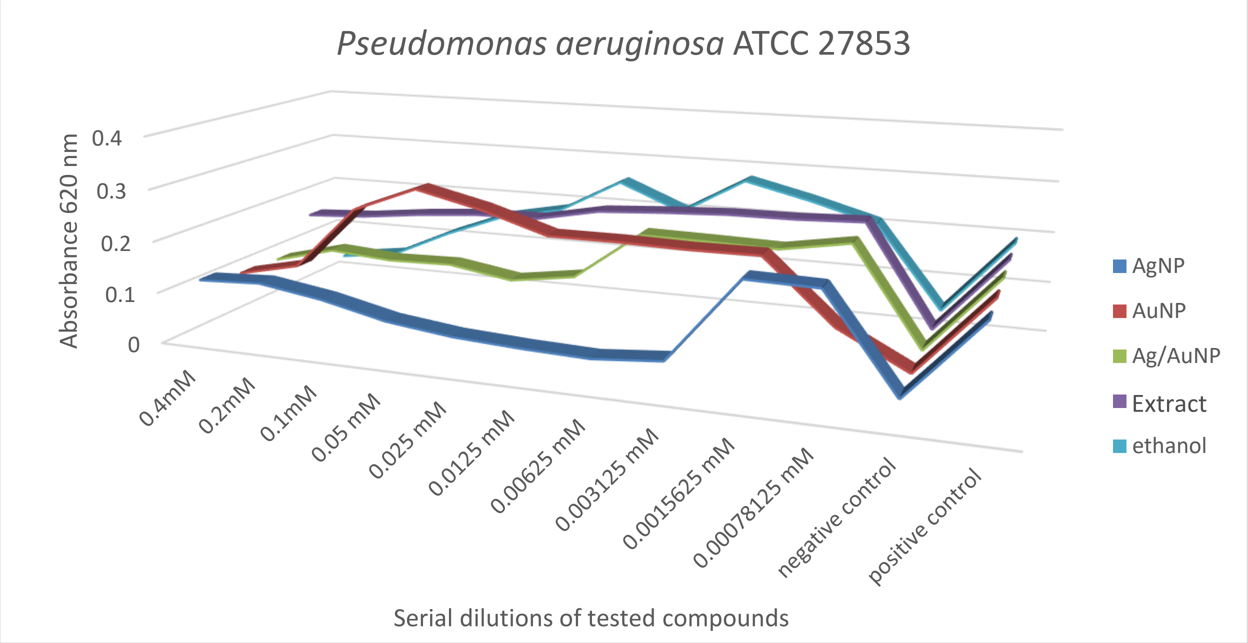

Figure S18. Graphic representation of minimal inhibitory concentration values after spectrofotometric reading of *P. aeruginosa* ATCC 27853 culture

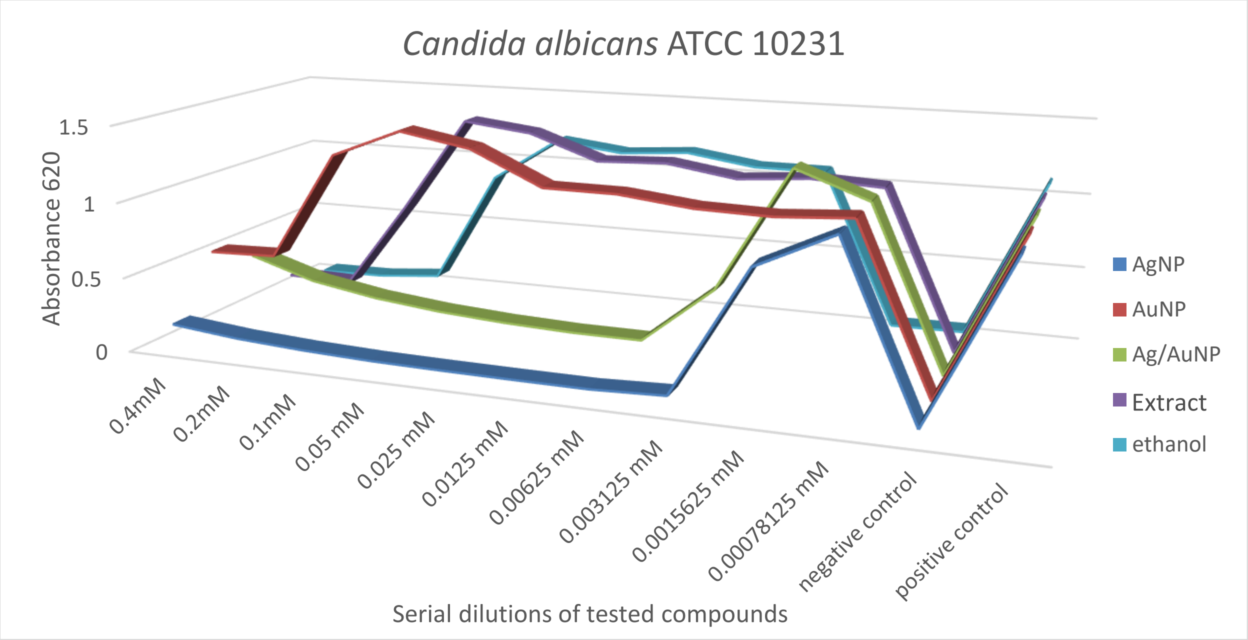

Figure S19. Graphic representation of minimal inhibitory concentration values after spectrofotometric reading of *C. albicans* ATCC 10231 culture

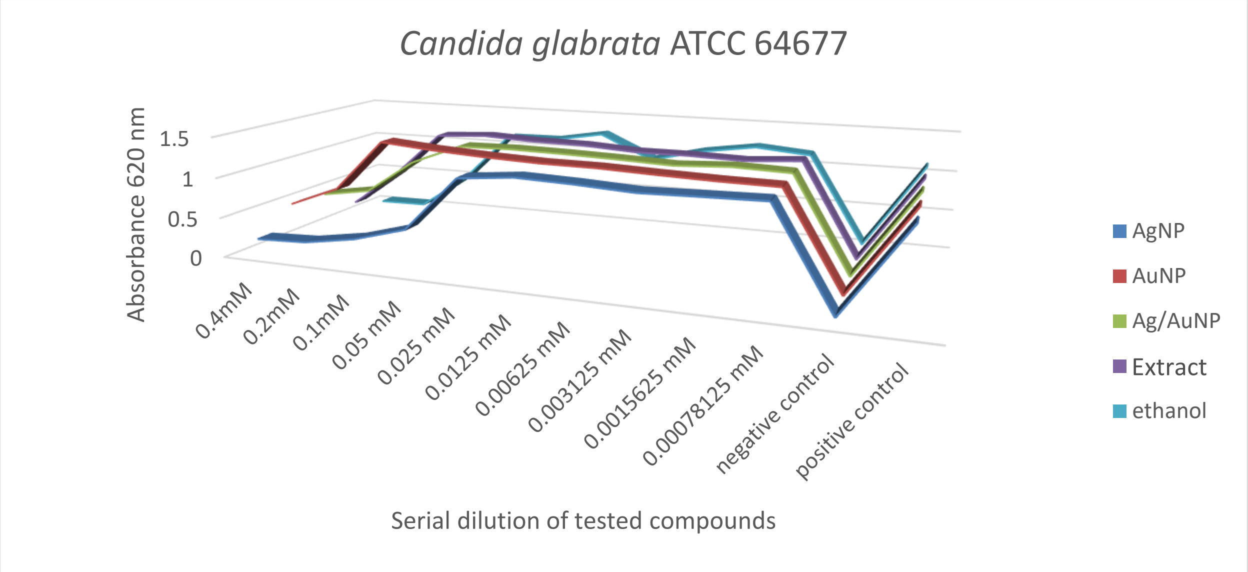

Figure S20. Graphic representation of minimal inhibitory concentration values after spectrofotometric reading of *C. glabrata* ATCC 64677 culture

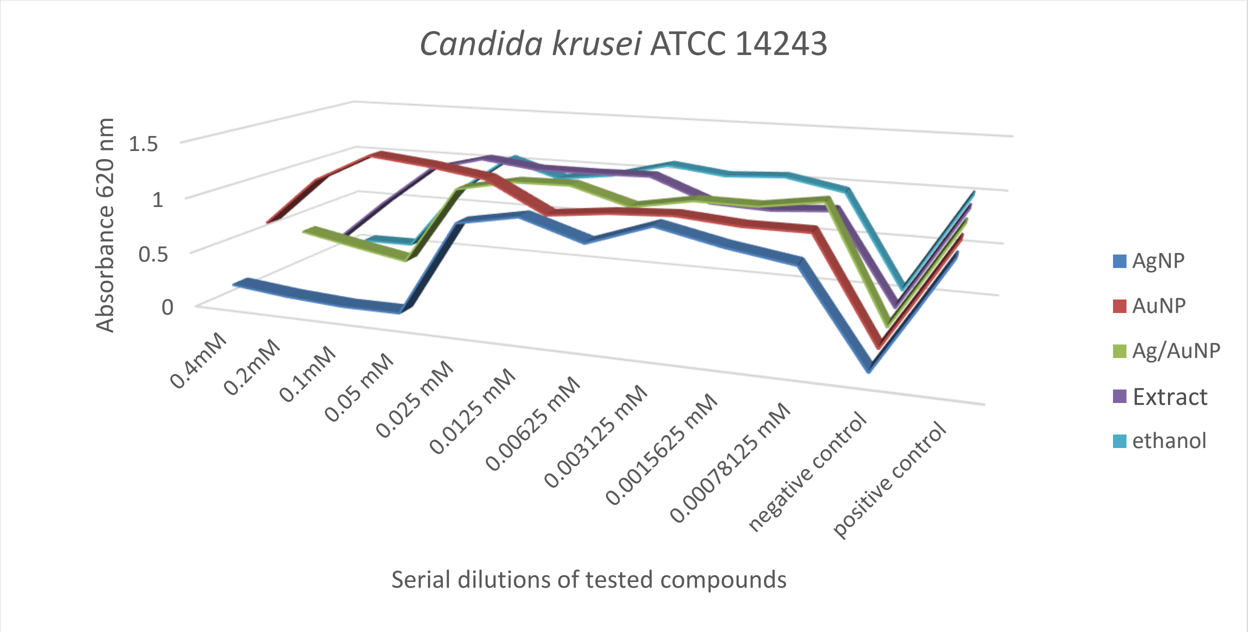

Figure S21. Graphic representation of minimal inhibitory concentration values after spectrofotometric reading of *C. krusei* ATCC 14243 culture

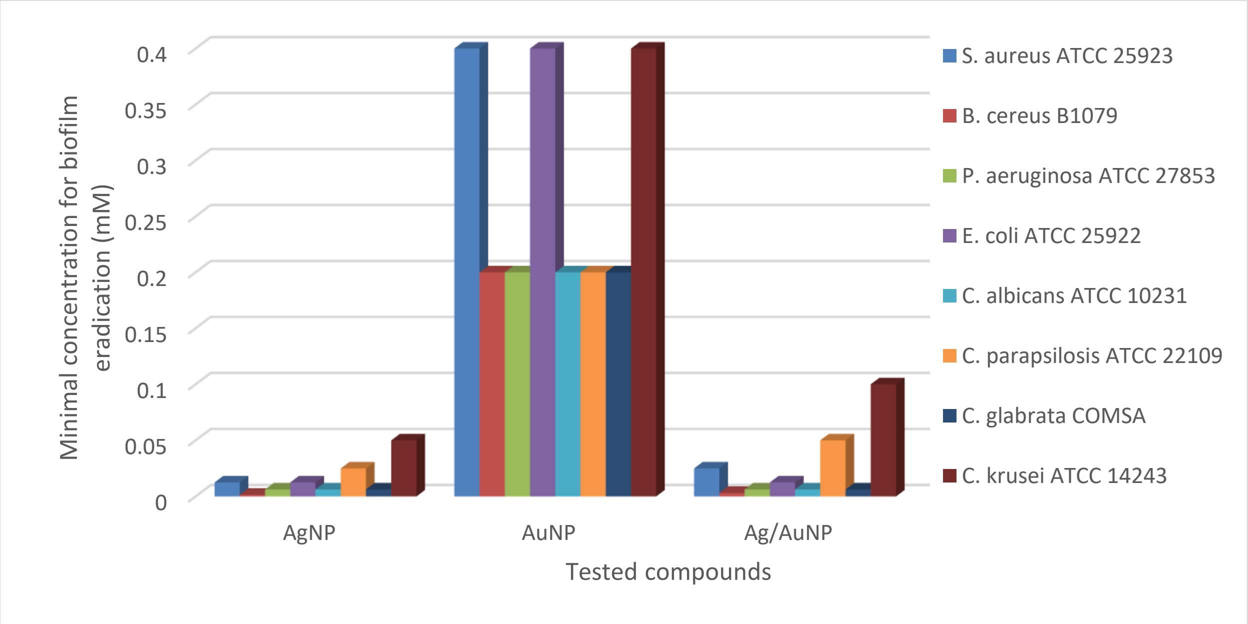

Figure S22. Graphic representation of minimal concentration of the tested compounds for biofilm eradication.

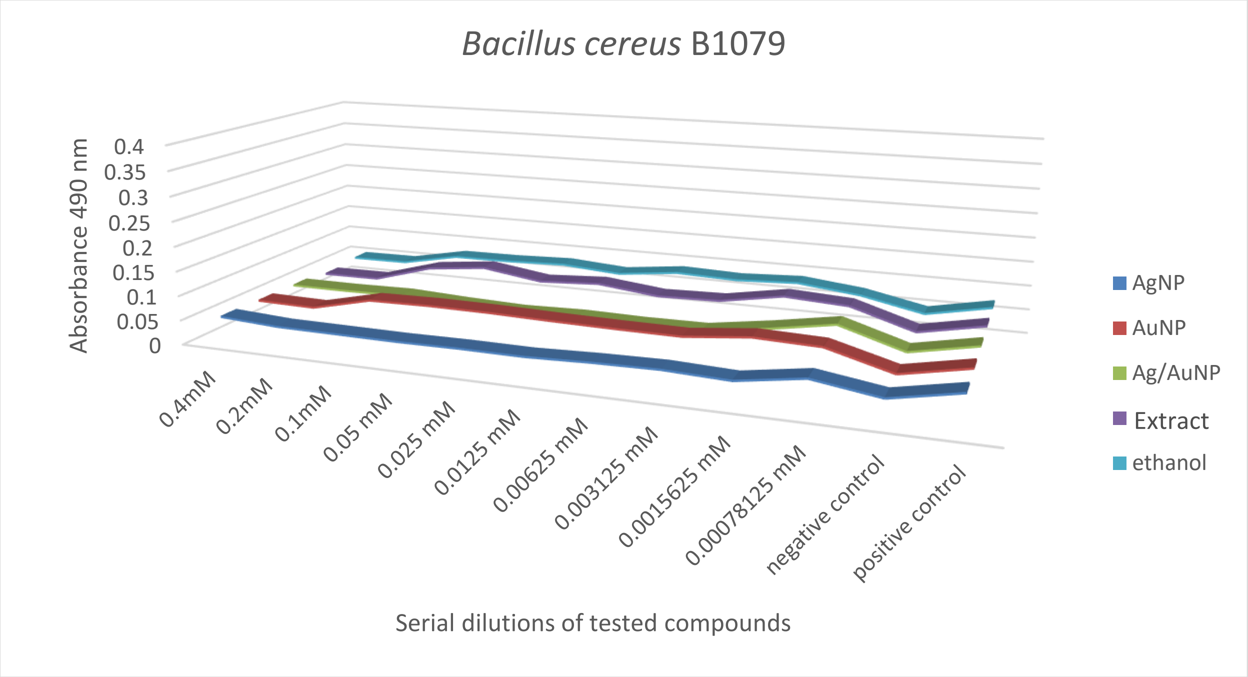

Figure S23. Graphic representation of minimal concentration for biofilm eradication values after spectrofotometric reading of *B. cereus* B1079 cell suspension, after crystal violet staining.

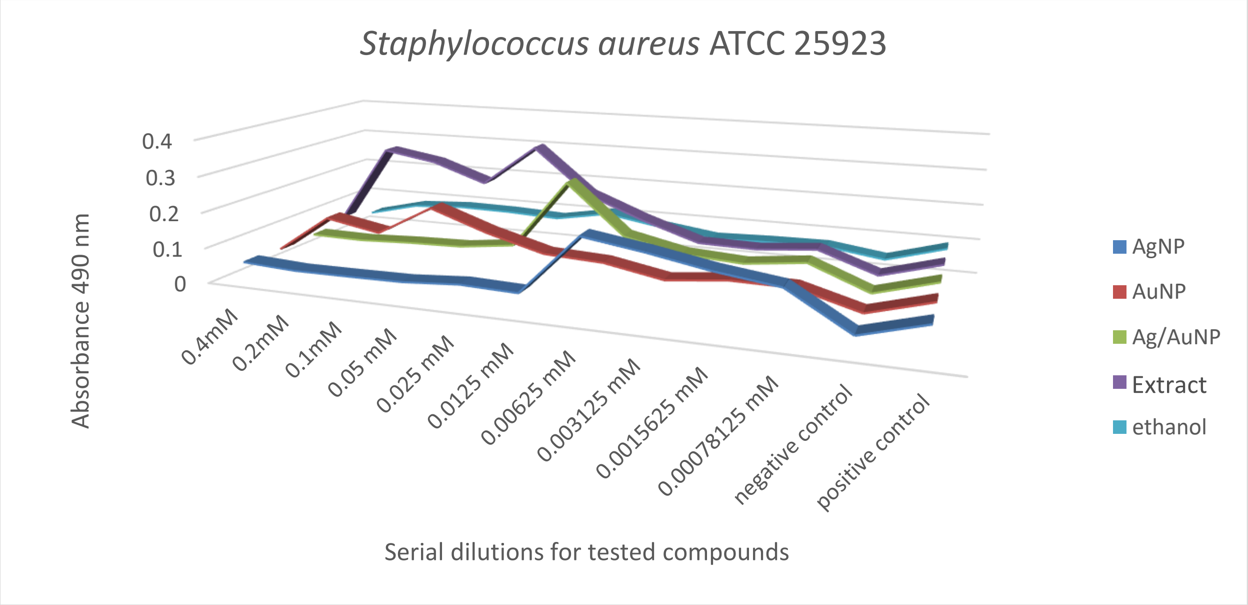

Figure S24. Graphic representation of minimal concentration for biofilm eradication values after spectrofotometric reading of *S. aureus* ATCC 25923 cell suspension, after crystal violet staining.

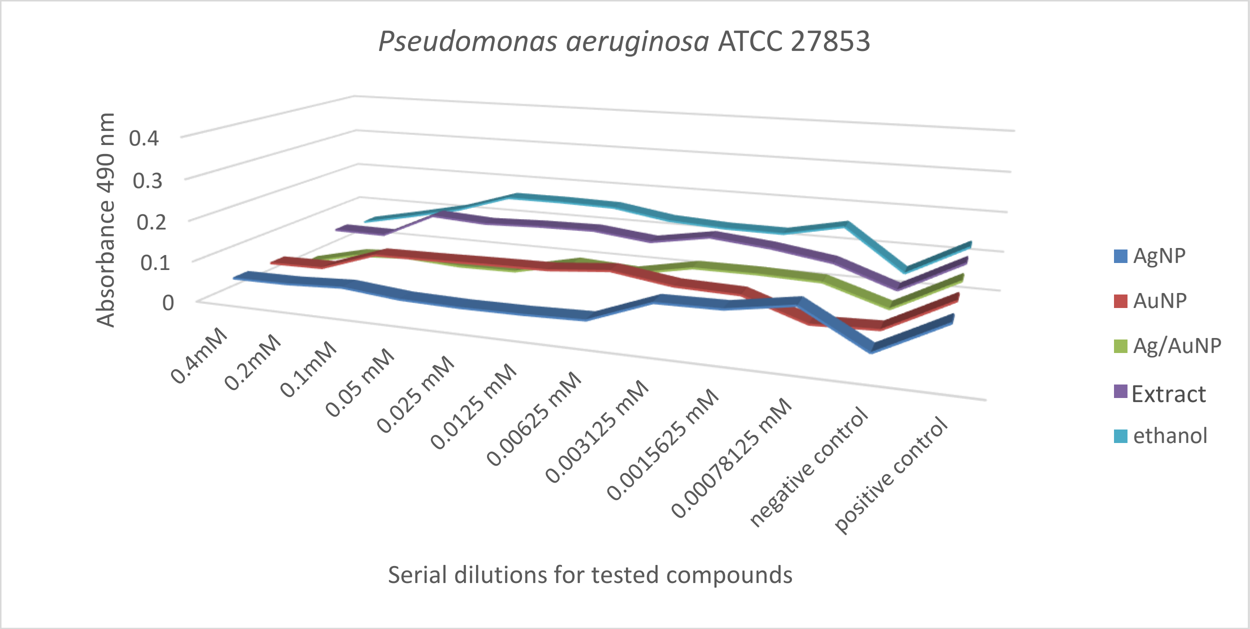

Figure S25. Graphic representation of minimal concentration for biofilm eradication values after spectrofotometric reading of *P. aeruginosa* ATCC 27853 cell suspension, after crystal violet staining.

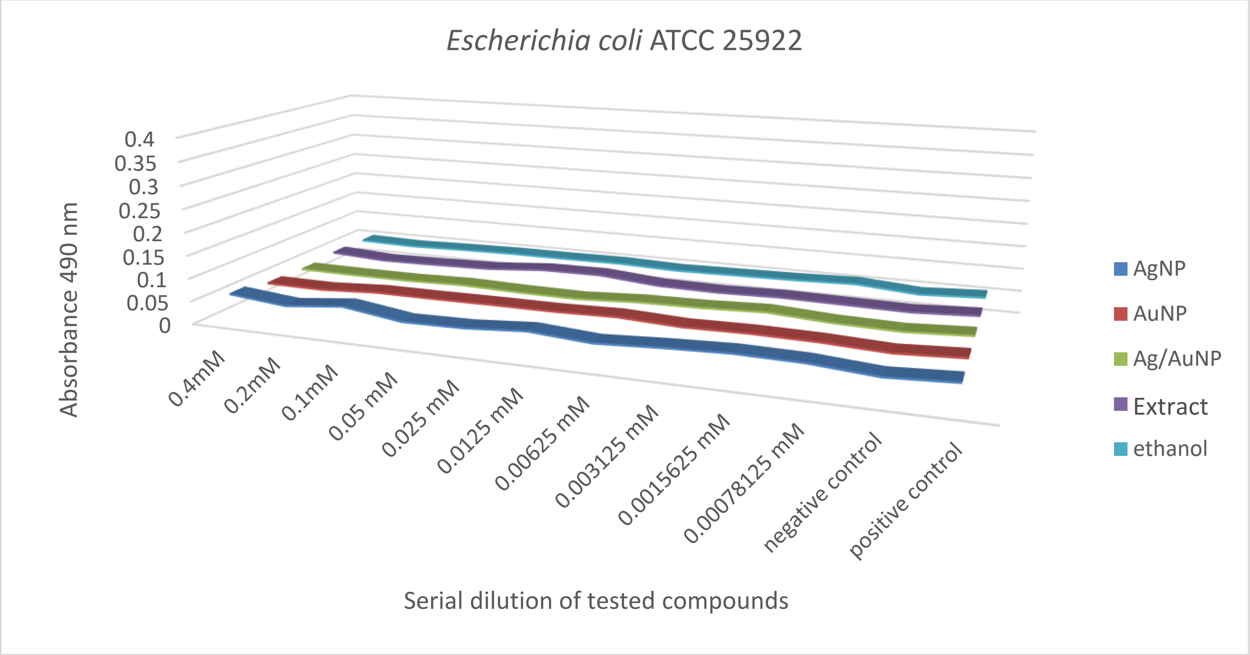

Figure S26. Graphic representation of minimal concentration for biofilm eradication values after spectrofotometric reading of *E. coli* ATCC 25922 cell suspension, after crystal violet staining.

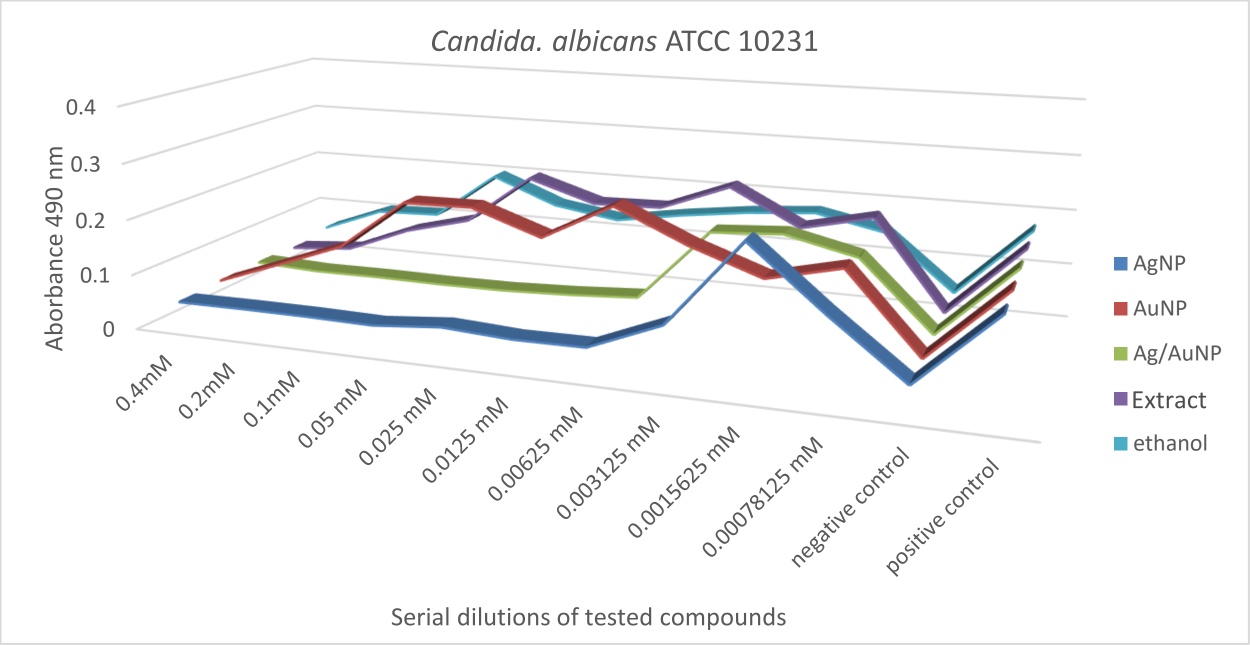

Figure S27. Graphic representation of minimal concentration for biofilm eradication values after spectrofotometric reading of *C. albicans* ATCC 10231 cell suspension, after crystal violet staining.

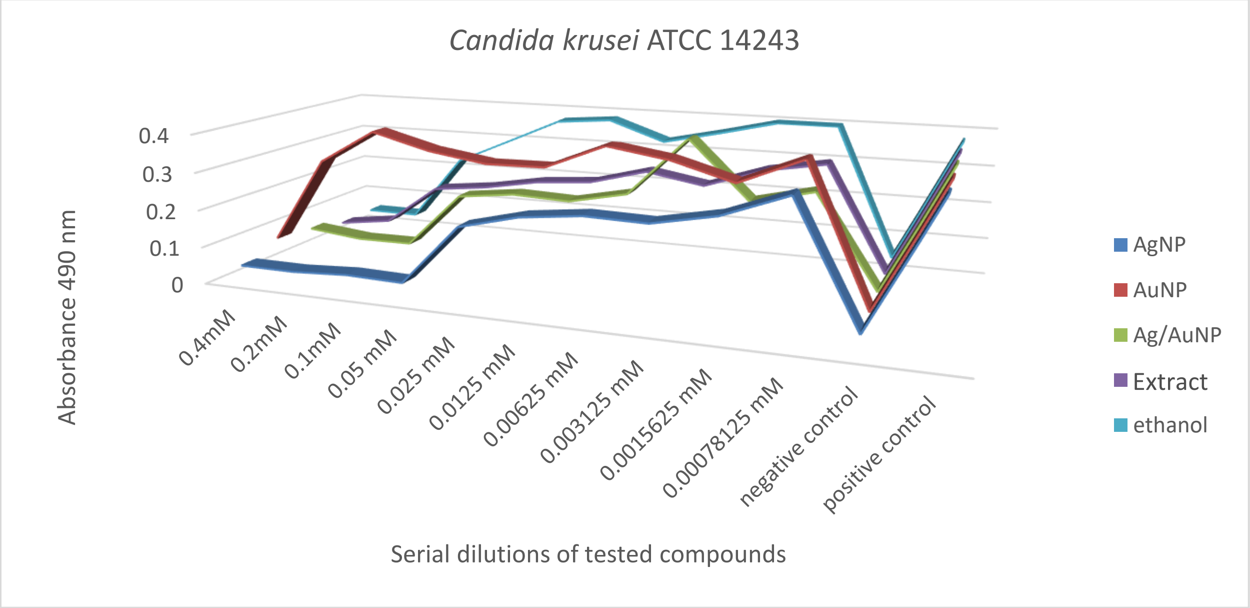

Figure S28. Graphic representation of minimal concentration for biofilm eradication values after spectrofotometric reading of *C. krusei* ATCC 14243 cell suspension, after crystal violet staining.
